# Barium titanate and lithium niobate permittivity and Pockels coefficients from megahertz to sub-terahertz frequencies

**DOI:** 10.1038/s41563-025-02158-1

**Published:** 2025-03-17

**Authors:** Daniel Chelladurai, Manuel Kohli, Joel Winiger, David Moor, Andreas Messner, Yuriy Fedoryshyn, Mohammed Eleraky, Yuqi Liu, Hua Wang, Juerg Leuthold

**Affiliations:** 1 Institute of Electromagnetic Fields (IEF), ETH Zurich, Zurich, Switzerland; 2 Integrated Devices, Electronics, and Systems (IDEAS) Group, ETH Zurich, Zurich, Switzerland; 3 Present Address: Zurich Instruments AG, Zurich, Switzerland

**Keywords:** Integrated optics, Nonlinear optics, Optoelectronic devices and components

## Abstract

The Pockels effect is essential for controlling optical signals at the highest speeds, particularly for electro-optic modulators in photonic integrated circuits. Lithium niobate (LN) and barium titanate (BTO) are two excellent Pockels materials to this end. Here we measure the Pockels coefficients and permittivity in LN and BTO over a continuous frequency range from 100 MHz to 330 GHz. These properties are constant across this frequency range in LN, but have a strong frequency dependence in BTO. Still, our measurements show that BTO has remarkable electro-optic properties compared with LN. Furthermore, we show how BTO devices can be designed with a flat electro-optic frequency response despite the Pockels coefficient dispersion. Finally, we expound our method for broadband characterization of these vital electro-optic properties, utilizing specialized integrated electro-optic phase shifters. Altogether, this work empowers the design of high-speed BTO devices and the development of new electro-optic materials.

## Abstract (web summary)

Pockels coefficients and permittivity are measured in barium titanate and lithium niobate from 100 MHz to 330 GHz and device geometries are proposed to maintain a constant electro-optic response in BTO devices.

## Main

The Pockels effect, or the linear electro-optic (EO) effect, is a widely used nonlinear optics phenomenon in which a near-instantaneous refractive-index change is induced by applying an electric field. The fast response is ideal for optical communications, where it enables EO modulator bandwidths to reach beyond 100 GHz (refs. ^1–4^). Pockels materials are used in the active region of these modulators to map an electrical signal onto an optical carrier. Pockels modulators find use in applications at lower speeds, too. For example, they are useful as microwave-optical transducers in quantum networks^5,6^, or as switches in programmable photonic circuits^7–9^. Pockels materials have also been demonstrated in reconfigurable metasurfaces^10,11^, which may be interesting for light detection and ranging, augmented reality and virtual reality applications. This wide range of applications requires the Pockels effect to work across an accordingly wide range of operating frequencies from megahertz to hundreds of gigahertz. It is, therefore, of high interest to know which EO materials can efficiently cover this range.

Ferroelectric materials like lithium niobate (LN) and barium titanate (BTO) are popular Pockels materials because their large optical refractive index is useful for waveguiding and their large Pockels coefficients are useful for EO effects. However, the frequency dependence of the Pockels coefficients in these materials has not been experimentally verified, to the best of our knowledge. Permittivity measurements are more commonly reported, yet measurements for thin films at frequencies above 10 GHz are scarce, especially for BTO. A simple and effective method for extracting the frequency-dependent permittivity and EO coefficients of a thin film directly from a device in photonic integrated circuits is highly desirable.

In this work, we experimentally verify the frequency responses of the permittivities as well as Pockels coefficients for LN and BTO covering the range from 100 MHz to 330 GHz. To this end, we introduce a method to characterize the Pockels coefficient and permittivity of thin films using specialized phase shifters in photonic integrated circuits. Furthermore, by comparing the frequency dependence of BTO’s permittivity with its Pockels coefficients, we show that the dispersion is linked to the *r*_42_ Pockels coefficient and permittivity along the crystalline *a* axis, *ε*_*a*_. Meanwhile, the *r*_33_ coefficient and *ε*_*c*_ remain constant above 1 GHz. We also present the interesting conclusion that for certain device geometries, the EO response in BTO devices can be kept constant with frequency despite the strong dispersion in its EO properties.

## Device for EO characterization

The EO characterization of thin films up to sub-terahertz frequencies presents numerous challenges. Unlike bulk crystals, it is not easy to place thin films in the path of a free-space interferometer. Integrated photonic devices must be used instead. Although integrated EO devices are not new, they are rarely operated at frequencies above 110 GHz because of bandwidth limitations. These bandwidth limitations arise from the RC time constant associated with charging the electrodes, radio-frequency (RF) attenuation along the length of the modulator, and/or velocity mismatch between the RF and optical signals^12^. The latter two effects can be mitigated if the length of the device is sufficiently short. This, however, comes with the trade-off of reduced EO efficiency and an RC limit will still exist. Additionally, it is difficult to generate signals at sub-terahertz frequencies with powers that are high enough to distinguish weak EO effects from noise. To measure the weak Pockels shift in bandwidth-limited or short, inefficient devices, it is necessary to have high optical input powers combined with low optical insertion loss. Taking the above points into consideration, the ideal EO characterization device should have a short length, low optical insertion loss and high EO efficiency per unit length.

**Fig. 1:**
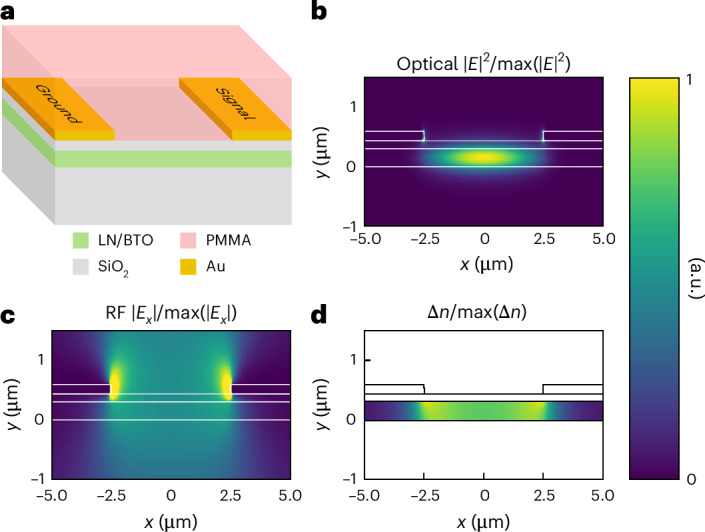
Integrated phase shifter used to measure permittivity and Pockels coefficients. **a**, Schematic of the cross-section of the phase shifter used for EO characterization. PMMA, polymethyl methacrylate. **b**, *E*-field profile of an optical mode in the phase shifter for *λ* = 1,550 nm. **c**, RF *E*-field profile when a 50-GHz electrical signal is applied to the electrodes. **d**, Spatially varying refractive-index change in the EO material due to the Pockels effect. The maximum Δ*n*/Δ*V* is on the order of 10^−5^ V^−1^ for LN and 10^−4^ V^−1^ for BTO. Note that the values in **b**–**d** are normalized to fit the same colour scale.

In pursuit of an ideal EO characterization device, we have developed a specialized phase shifter that is based on hybrid gap plasmon waveguides^13,14^. Compared with previous demonstrations, we replace the silicon layer of the hybrid gap plasmon waveguide structure with an EO material. In this way, the optical mode is confined within an EO material even when the gap between the electrodes is large and the optical losses are small. A cross-sectional schematic of the device is shown in Fig. 1a. A thin film of an EO material like LN or BTO on a buried oxide is the primary waveguiding layer. Figure 1b shows the optical mode’s electric-field intensity profile at a wavelength of 1,550 nm. The electrodes guide the optical mode similar to nanoscale plasmonic slot modulators, but with a much larger spacing between the electrodes to reduce optical propagation losses. An electrical signal applied to the electrodes results in the RF electric-field profile shown in Fig. 1c. The presence of this field in the EO material causes a refractive-index change due to the Pockels effect according to equation (1)^15^:

1 $$\Delta n=-\frac{1}{2}{n}_{0}^{3}{r}_{{\rm{eff}}}E,$$

where *n*_0_ is the material’s unperturbed refractive index, *r*_eff_ is the effective Pockels coefficient and *E* is the RF electric field in the material. Figure 1d shows this spatially varying refractive-index change Δ*n*. The visualizations shown in Fig. 1 are qualitative. The values in each figure are normalized such that the maximum is unity. In reality, the true values depend on the optical and RF powers, as well as the permittivity and Pockels coefficients, which can vary widely between EO materials.

There are several benefits to using this kind of device for EO characterization. First, it covers the aspects of the ideal EO characterization device discussed above. The phase shifters are efficient. The optical field is mostly confined to the EO layer and, more importantly, also within the region that undergoes a refractive-index change. Because the devices are efficient, their lengths can be reduced to only tens of micrometres (25 µm for BTO or 50 µm for LN) and still provide useful modulation. With such short devices, the capacitance is small and the RC bandwidth limitation is pushed to frequencies in the hundreds of gigahertz. There is no need for impedance matching or RF terminations, and the effects of a velocity mismatch—or walk-off—between the RF and optical signals is negligible. The short devices also have negligible on-chip insertion losses, which helps with measuring Pockels coefficients despite weak EO signals. The total insertion loss is approximately 0.2 dB based on mode simulations and supported by our previous experiments^16^. For a phase shifter with a 50-µm length and 5-µm electrode separation, propagation losses account for roughly 0.1 dB (20 dB cm^–1^), with the remaining 0.1 dB caused by reflection losses at the transitions to and from the device. The phase shifter also benefits from a straightforward fabrication process. As described in Methods, both the structure and the fabrication process leave little uncertainty with respect to dimensions and alignment.

## Permittivity

**Fig. 2:**
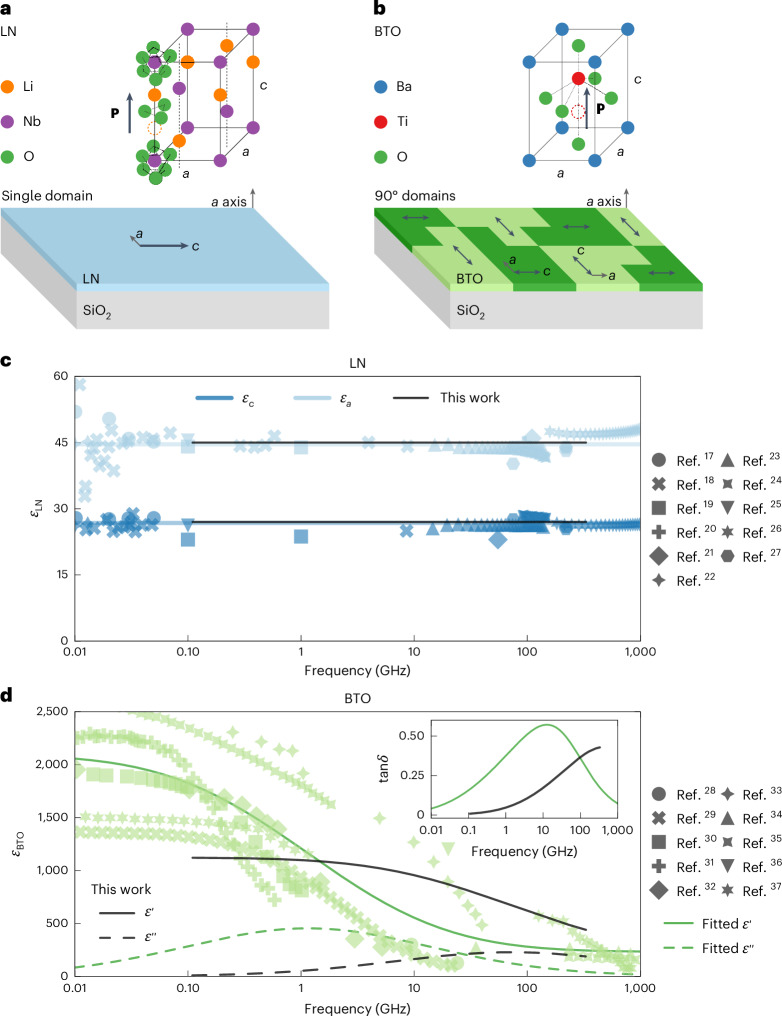
Crystal structure and permittivity of LN and BTO. **a**,**b**, Crystal structures of LN (**a**) and BTO (**b**) and how they are oriented as thin films. In each unit cell, the spontaneous polarization **P** is marked with the black arrow. The ‘empty’ atoms with the dashed outline represent the hypothetical positions of the Li (orange) or Ti (red) ions if the polarization was flipped. In the depiction of thin films, the black arrows are also used to represent the spontaneous polarization. Both have an in-plane *c* axis. LN has a single domain, with only one polarization direction (indicated by the single black arrow). BTO has a multidomain structure in which the *c* axes of the neighbouring domains are oriented at 90° to each other (indicated by the multiple black arrows and differently shaded regions). For BTO, the black arrows are double-sided to represent the fact that the raw film might have **P** in either direction. **c**, LN permittivity data along the *a* axis (light blue) and *c* axis (dark blue) collected from the literature^17–27^. The blue solid lines represent a linear fit to the literature data. The black lines represent the values produced from the model in this work. **d**, BTO permittivity data (light green) collected from the literature^28–37^. The green solid and dashed lines are the real and imaginary parts, respectively, of the Debye model from equation (2) fitted to the literature data. The permittivity values extracted from the measurements of the RF-sputtered BTO of this work are plotted in black. The inset shows the loss tangent tan*δ* = *ε*″/*ε*′ for the literature fit and the data from this work.

There are stark differences between the permittivities of LN and BTO as a function of frequency. In this section, we discuss the differences between thin films of the two materials and present the measurements of their permittivities alongside the available literature data. In Supplementary Note 1, we elaborate on the origins of permittivity in ferroelectrics. The crystal structures of both LN and BTO lead to large anisotropies in their respective permittivity. Figure 2a shows a sketch of the trigonal unit cell for LN belonging to the 3*m* point group, and Fig. 2b shows the same for BTO’s tetragonal unit cell belonging to the 4*mm* point group. For clarity, most of the oxygen atoms in the LN unit cell have been omitted. The direction of the displacement of cations along the *c* axis determines the direction of spontaneous polarization (Fig. 2, black arrows).

Thin films of LN and BTO exhibit some differences in their domain structures. First, LN is typically produced as a single-domain film in which the polarization of every unit cell is aligned in the same direction. This is indicated with the single arrow in the plane of the LN film (Fig. 2a). By contrast, a raw BTO thin film can have domains with the ferroelectric polarization randomly oriented in one of four 90° orientations (Fig. 2b, double-sided arrows).

The permittivity measurements here are presented differently for each material because of their different domain structures. Figure 2c,d shows the measured permittivity data collected from various literature sources for LN^17–27^ and BTO^28–37^, respectively. The permittivity in most studies on LN can be separately measured along the *a* and *c* axes because of its stable single-domain structure. The values for LN in the literature (Fig. 2c) are, therefore, categorized by *ε*_*a*_ (light blue) and *ε*_*c*_ (dark blue). The permittivity of LN can be described with a constant model in this frequency range. The solid lines in Fig. 2c represent the mean values of *ε*_*a*_ = 45 and *ε*_*c*_ = 27 in the literature. These values are also used in the analysis of our own measurements to validate our method for measuring the permittivity, which is described in further detail in Methods and Supplementary Note 2. Our work confirms these values, as indicated by the black lines in Fig. 2d.

In contrast to the permittivity measurements of LN, the typical mixed-domain structure of BTO makes it difficult to distinguish between *ε*_*a*_ and *ε*_*c*_. Instead, the permittivity is often reported without reference to a particular crystal axis. The literature data for BTO in Fig. 2d (light green) are, therefore, the effective permittivity with contributions from both *ε*_*a*_ and *ε*_*c*_; our own thin-film measurements are plotted in black.

The BTO permittivity has considerable variability among the reported results and cannot be described by a linear fit. From a high-level perspective, the variability is a result of differences in the sample quality and crystal growth techniques^31^. Some sources measure ceramics produced from powders^28–30,32,35,37^; others measure bulk crystals produced by top-seeded solution growth^30,31^; and more recent reports measure thin films grown by metal–organic vapour deposition^33,34^, molecular-beam epitaxy^36^ and chemical solution deposition^38,39^. From a microscopic perspective, samples can have substantial variation in the quantity of defects, domain structures and poling of domains. We discuss how the domain structure and poling affects the permittivity in Supplementary Note 1.

The role of defects is especially relevant because our BTO films are grown by a recently developed epitaxial RF-sputtering method^40,41^. This method is suggested to minimize defects as it starts from a stoichiometric BTO target, resulting in a stoichiometric thin film. Other epitaxial deposition methods like molecular-beam epitaxy or metal–organic chemical vapour deposition require a careful balance of precursors and still result in crystal defects such as oxygen vacancies that require annealing to be mitigated^42,43^. Defects in the crystal are attributed to a relaxation that falls in the range from hundreds of megahertz to gigahertz frequencies^30,31^. In particular, impurities and oxygen vacancies caused by non-stoichiometric crystal growth can drastically affect the strength and the central frequency of this relaxation^30,31^. A second relaxation in the range of 50 GHz or higher is associated with the Ti-ion hopping between sites along the *a* axis in the unit cell and is the proposed origin of the large permittivity along the *a* axis of BTO^31,44,45^.

We use a Debye model with a normal distribution of logarithmic relaxation frequencies^44^ to describe the permittivity of our BTO thin film as well as the collection of values from the literature. It takes the following form:

2 $$\varepsilon ^{\prime} (f)+{\rm{j}}\varepsilon ^{\prime\prime} \left(f\right)={\varepsilon }_{\infty }+\sum _{\rm{i}}\Delta {\varepsilon }_{\rm{i}}\int {\mathscr{N}}\left(\gamma ,{\gamma }_{{0}_{\rm{i}}},{\sigma }_{\rm{i}}\right)\frac{\gamma }{\gamma -{\rm{j}}{f}}{\rm{d}}\gamma$$

$${\mathscr{N}}\left(\gamma ,{\gamma }_{{0}_{\rm{i}}},{\sigma }_{\rm{i}}\right)=\frac{1}{{\sigma }_{\rm{i}}\sqrt{2\uppi }}{{\rm{e}}}^{-\frac{1}{2}{\left(\frac{\left(\log \gamma -\log {\gamma }_{{0}_{\rm{i}}}\right)}{{\sigma }_{\rm{i}}}\right)}^{2}}.$$

The summation accounts for the potential to have multiple relaxations as described above (for example, i = 1 for a defect-related relaxation, i = 2 for the Ti-ion relaxation, i = 3 for domain poling and so on). Here *γ* is the relaxation frequency, Δ*ε*_i_ is the relaxation strength and *ε*_∞_ is the constant permittivity that the model approaches at the high-frequency limit. The normal distribution of logarithmic relaxation frequencies ${\mathscr{N}}\left(\gamma ,{\gamma }_{{0}_{\rm{i}}},{\sigma }_{\rm{i}}\right)$ is described by its centre relaxation frequency ${\gamma}_{{0}_{\rm{i}}}$ and its width in log space *σ*_i_, and j is the imaginary unit. Table 1 lists the fitted parameters for the data shown in Fig. 2d, where the Debye model has been plotted for our measurements (black line) and the collection of literature data (green line). We obtained the best fit for the trend in the literature data with only one relaxation. A high-frequency relaxation related to the Ti ion could not be fit because the data in the literature at these frequencies are too sparse. For our data, the model gives the best fit with only a high-frequency relaxation. This result implies that our BTO film has minimal defects, as expected for RF-sputtered films. Additionally, our BTO is partially poled to reduce antiparallel domains, which reduces the permittivity especially at lower frequencies^46^.

**Table 1:** Fitted parameters of the Debye model for the BTO permittivity data

Permittivity data	*γ*_0_ (GHz)	*σ*	Δ*ε*	*ε*_∞_
Literature	1.15 ± 0.30	0.92 ± 0.20	1,862 ± 149	231 ± 79
This work	72.6 ± 2.3	0.87 ± 0.02	905 ± 26	231^a^

The Debye model is provided in equation (2) and the BTO permittivity data are shown in Fig. 2d. The uncertainty values are the standard errors of the fits. Note also that *σ* represents the width of the distribution of log*γ*. ^a^This value is fixed to the one from the literature data because our equipment limits us to 330 GHz. This is supported by multiple sources^34,35,37^ that report permittivity up to 1 THz.

We also show the imaginary parts of permittivity *ε*″ (Fig. 2d (inset), dashed lines). *ε*″ is related to the dielectric loss in the material.

Because we are only able to measure up to 330 GHz, we fix *ε*_∞_ to the value from the literature data in which measurements from 330 GHz to 1 THz and beyond are available from multiple sources^34,35,37^. The literature data have a much clearer consensus at terahertz frequencies than in the megahertz and gigahertz ranges.

## Pockels coefficient

Here we discuss the Pockels effect in LN and BTO. The Pockels tensors for both materials using the reduced Voigt notation are given as^47^

3 $${r}_{{{\rm{ij}}},{\rm{LN}}}=\left[\begin{array}{ccc}0 & -{r}_{22} & {r}_{13}\\ 0 & {r}_{22} & {r}_{13}\\ \begin{array}{c}0\\ 0\\ \begin{array}{c}{r}_{42}\\ {r}_{22}\end{array}\end{array} & \begin{array}{c}0\\ {r}_{42}\\ \begin{array}{c}0\\ 0\end{array}\end{array} & \begin{array}{c}{r}_{33}\\ 0\\ \begin{array}{c}0\\ 0\end{array}\end{array}\end{array}\right],{r}_{{{\rm{ij}}},{\rm{BTO}}}=\left[\begin{array}{ccc}0 & 0 & {r}_{13}\\ 0 & 0 & {r}_{13}\\ \begin{array}{c}0\\ 0\\ \begin{array}{c}{r}_{42}\\ 0\end{array}\end{array} & \begin{array}{c}0\\ {r}_{42}\\ \begin{array}{c}0\\ 0\end{array}\end{array} & \begin{array}{c}{r}_{33}\\ 0\\ \begin{array}{c}0\\ 0\end{array}\end{array}\end{array}\right].$$

**Fig. 3:**
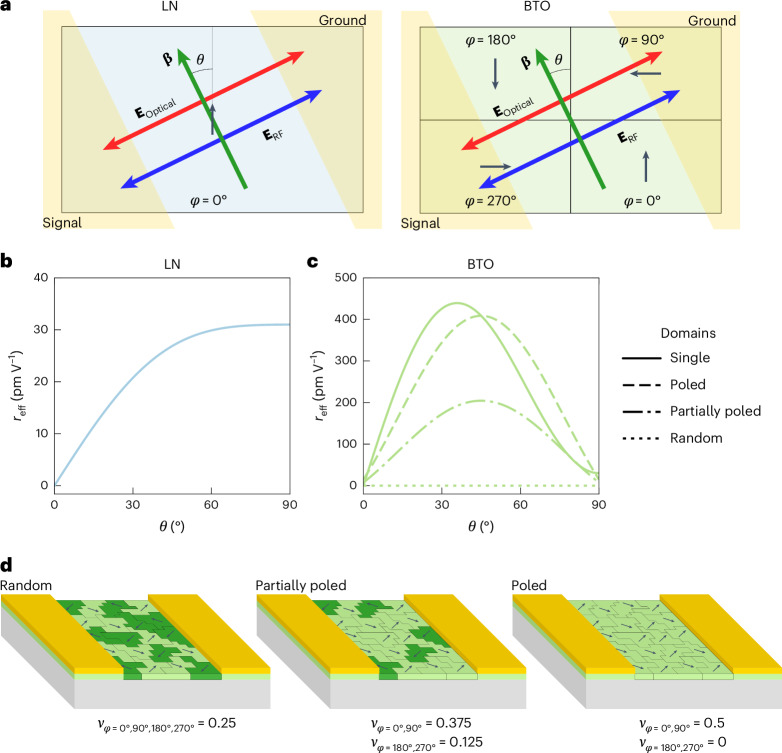
Phase-shifter orientation relative to crystal domains and the effective Pockels coefficient. **a**, Schematic depicting the phase-shifter orientation relative to the domain orientations in the LN (left) and BTO (right) thin films. The orientation of the ferroelectric polarization domains is shown by the black arrows and denoted by angle *φ*. The LN thin film only has a single polarization direction of *φ* = 0°, whereas BTO has four possible orientations, namely, *φ* = 0°, 90°, 180° and 270°. The areas shaded in gold represent the signal and ground electrodes, which also serve as optical waveguides. The phase shifters are oriented such that the propagation direction (green arrow) is at an angle *θ* relative to the crystal *c* axis. The optical (red arrow) and RF (blue arrow) electric fields are both polarized perpendicular to the propagation direction. **b**,**c**, Analytically calculated effective Pockels coefficients according to equation (4) for LN (**b**) and BTO (**c**). The solid line shows the case in which all the domains are oriented in the same direction (*ν*_*φ*=0°_ = 1). The dashed and dotted lines show the case for films with domains oriented at 90° to each other, in which the domains are fully poled (dashed), partially poled (dot–dashed) or randomly distributed (dotted). **d**, Illustrations of the domain polarizations in BTO for the three cases of poling along with their domain fractions *ν*_*φ*_. Domains with the preferred polarization direction are coloured in light green, and the antiparallel domains are coloured in dark green.

The magnitude of the refractive-index change depends on the orientation of both optical (**E**_Optical_) and RF (**E**_RF_) fields relative to the crystal orientation. The effective Pockels coefficient *r*_eff_ is the sum of the contributions from each element in the Pockels tensor depending on the orientations of **E**_Optical_ and **E**_RF_ relative to the crystal axes^48,49^. Figure 3a illustrates these orientations in the context of the phase shifters in this work. In this configuration, the analytical equation for the effective Pockels coefficient in both LN and BTO takes the following form, which is derived in Supplementary Note 3.

4 $${r}_{{\rm{eff}}}\left(\theta \right)=\sum _{\varphi }{\nu }_{\varphi }\left({\sin }^{2}\left(\theta +\varphi \right)\sin \left(\theta +\varphi \right)\left({r}_{13}+2{r}_{42}\right)+{r}_{33}{\sin }^{3}\left(\theta +\varphi \right)\right)$$

Following the convention in a previous analysis^43^, here we have also introduced the terms *φ* and *ν*_*φ*_ to account for the domain orientation and the fraction of domains with a particular orientation, respectively. *φ* can take one of four possible values (0°, 90°, 180° and 270°) representing the direction of ferroelectric polarization in the plane of the thin film. *ν*_*φ*_ is the relative fraction of domains in the thin film with orientation *φ* and can take a value between 0 and 1, with Σ_*φ*_*ν*_*φ*_ = 1. To demonstrate the effects of different domain distributions, we compare *r*_eff_ as given by equation (4) in Fig. 3b for LN and Fig. 3c for BTO for four cases. The curves were calculated using literature data for the clamped Pockels coefficients measured near 10 MHz for LN^25^ and BTO^50^. The single-domain case (Fig. 3c, solid line; *ν*_*φ*=0°_ = 1) applies to the LN films but is also plotted for a hypothetical single-domain BTO film. For LN, *r*_eff_ is maximum when **E**_Optical_ and **E**_RF_ are parallel to the *c* axis (*θ* = 90°) because *r*_33_ is the largest Pockels coefficient in LN. The multidomain structure of BTO thin films results in a maximum *r*_eff_ when *θ* = 45°. This is shown (Fig. 3c) for three cases in which a film is poled (dashed line), partially poled (dash–dotted line) or random (dotted line). Figure 3d illustrates these three cases, where the black arrows represent the polarization direction and the light-green domains are polarized in the preferred direction. The maximum value of *r*_eff_ is achieved for the poled case when all the ferroelectric polarizations in the domains are aligned in a preferred direction. When domains have polarizations in opposite directions, the net Pockels shift will be zero, as is the case for the random film. The frequency dependence remains approximately the same regardless of the domain distribution because it originates from the ions in the BTO unit cell. Polarizations can be aligned preferentially using an external d.c. bias; however, in our measurements, this was not possible at the highest frequencies due to the limitations of our instruments. The partially poled case is the most realistic case for the BTO films in our measurements with domain fractions that were reported for similar thin films^43^. This state is reached after poling the film with a d.c. bias and then removing the bias before performing EO measurements. Further discussions on this poling procedure and the domain fractions are given in Supplementary Note 3 and Methods, respectively.

**Fig. 4:**
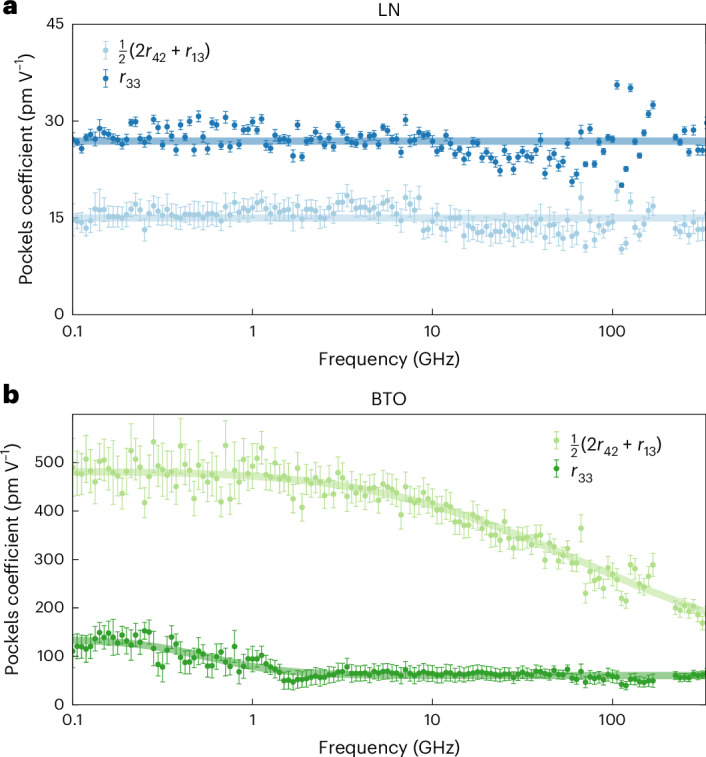
Pockels coefficients of LN and BTO. **a**, Pockels coefficient measurements for LN, with *r*_33_ in dark blue, *r*_42_ in light blue and linear fits to the data in solid lines. The fitted values are *r*_33_ = 26.9 pm V^–1^ and 1/2(*r*_13_ + 2*r*_42_) = 15.0 pm V^–1^. **b**, Pockels coefficient measurements for BTO, with *r*_33_ in dark green, *r*_42_ in light green and model fits to the data in solid lines. The *r*_42_ data are fit to a Debye model with *γ*_0_ = 565 MHz, *σ* = 0.06, *S*_R_ = 75 pm V^–1^ and *r*_∞_ = 60 pm V^–1^. The 1/2(*r*_13_ + 2*r*_42_) value is fit using equation (5), where *ε* is calculated from the Debye model of equation (2) using the same coefficients as the measured permittivity (Table 1). The data points are the result of fitting the measured *r*_eff_ data from *n* = 15–20 devices to equation (4), as described in Supplementary Information. The error bars represent two standard errors of the fit. Note that typically 2*r*_42_ ≫ *r*_13_ so that *r*_13_ + 2*r*_42_ ≈ 2*r*_42_. We, therefore, plot this parameter as 1/2(*r*_13_ + 2*r*_42_) ≈ *r*_42_.

Effective Pockels coefficients of the LN and BTO films were extracted from phase modulation measurements using the measurement setup and analysis procedure described in Methods. *r*_eff_ was measured for different phase shifters oriented at varying angles *θ* to the substrate. The set of *r*_eff_(*θ*) data points at each frequency was to fit equation (4), with the domain fractions corresponding to the partially poled case. The fitting process yields values for *r*_13_ + 2*r*_42_ and *r*_33_, as explained in further detail in Supplementary Note 3. Figure 4 shows the results of this measurement and fitting process for both LN (blue) and BTO (green) across all the measured frequencies. In both cases, the error bars represent ±two standard errors of the fit.

The LN Pockels coefficients are presented in Fig. 4a. LN is a good reference material because its EO properties are well known and it is not expected to show any dispersion in the measured frequency range. Indeed, the data for both *r*_33_ (dark blue) and *r*_13_ + 2*r*_42_ (light blue) are flat over three orders of magnitude in frequency. The solid lines are linear fits to the measured data, with *r*_33_ = 26.9 pm V^–1^ and 1/2(*r*_13_ + 2*r*_42_) = 15.0 pm V^–1^. We note that the lower values compared with the literature^25^ are probably due to the fact that we have used an optical wavelength of 1,550 nm instead of 633 nm, which has been shown to influence the Pockels coefficient in ferroelectrics^50^.

BTO’s Pockels coefficients are presented in Fig. 4b. The *r*_33_ data (dark green) show a dispersion step at a few hundreds of megahertz, whereas the *r*_13_ + 2*r*_42_ data (light green) show strong dispersion above 10 GHz. The solid lines represent the same type of Debye model that was used to model the BTO permittivity. For *r*_33_, the data are fitted with parameters *γ*_0_ = 565 MHz, *σ* = 0.06, *S*_R_ = 75 pm V^–1^ and *r*_∞_ = 60 pm V^–1^ (analogous to *ε*_∞_). The *r*_33_ value of 125 pm V^–1^ at 100 MHz is in close agreement with recent measurements of similar films for frequencies below 10 MHz (ref. ^41^). In Methods, we show that the Pockels coefficient can be expressed as a function of the RF permittivity through Miller’s rule^51^:

5 $$r\approx 2\delta \frac{{\left({n}_{0}^{2}-1\right)}^{2}}{{n}_{0}^{4}}\left(\varepsilon -1\right),$$

where *n*_0_ = 2.26 is the optical refractive index for BTO at 1,550 nm and *δ* is Miller’s coefficient. The *r*_13_ + 2*r*_42_ model (Fig. 4b) is from equation (5), where *ε* is calculated from the coefficients listed in Table 1. The best fit was found to be *δ* = 0.327 ± 0.002 pm V^–1^. This gives values for 1/2(*r*_13_ + 2*r*_42_) of 481 pm V^–1^ at 100 MHz and 191 pm V^–1^ at 330 GHz. Equation (5) is in excellent agreement with the measurements and lends credence to the empirical Miller’s rule. Much like permittivity, BTO’s *r*_42_ coefficient is remarkably large at lower frequencies but decreases substantially across the measured frequency range. Given the link between the first- and second-order susceptibilities, the measured Pockels coefficients imply that the permittivity dispersion is related to the same underlying phenomenon as the *r*_13_ + 2*r*_42_ dispersion. In fact, these results suggest that the decrease in the overall permittivity of the film can be entirely attributed to a decrease in *ε*_*a*_. Because the frequency response of the *r*_33_ coefficient is flat above 1 GHz, it would be reasonable to assume that the permittivity along the BTO *c* axis is probably constant across this frequency range, too.

## Frequency dependence of the Pockels shift in BTO devices

The results above provoke the following question: if BTO’s *r*_42_ decreases with frequency, is it unfavourable to BTO’s suitability for high-speed devices? In this section, we show that the answer depends on the device geometry by comparing BTO in photonic and plasmonic structures.

**Fig. 5:**
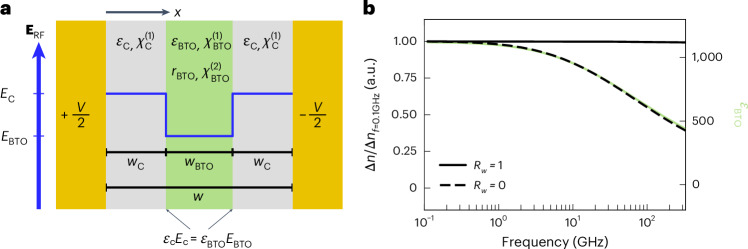
Frequency dependence of the Pockels shift in a device. **a**, Schematic of a hypothetical device with a potential difference *V* across two electrodes separated by a width *w*; a cladding material (grey) with permittivity *ε*_C_, susceptibility ${\chi }_{{\rm{C}}}^{\left(1\right)}$ and width *w*_C_; and an EO material (green) with permittivity *ε*_BTO_, susceptibility ${\chi }_{{\rm{BTO}}}^{\left(1\right)}$ and width *w*_BTO_. The blue line marks how the boundary condition at the bottom of the diagram affects the electric-field strength in each material assuming *ε*_BTO_ > *ε*_C_. **b**, Refractive-index change in the BTO material according to equation (6) as a function of frequency for varying ratios *R*_*w*_ with *ε*_C_ = 4. The measured permittivity of BTO in this work (Table 1) was used as the input for *ε*_BTO_ and is plotted in green. Δ*n* is normalized to its low-frequency value at 100 MHz to highlight the frequency dependence for each case.

To start this discussion, we introduce the simplistic, hypothetical structure shown in Fig. 5a. Two metal electrodes with a potential difference *V* are placed on each side of a BTO layer with width *w*_BTO_ and RF permittivity *ε*_BTO_ = 1 + ${\chi }_{{\rm{BTO}}}^{\left(1\right)}$. The electrodes are separated from the BTO layer by a cladding material with width *w*_C_ and RF permittivity *ε*_C_ = 1 + ${\chi }_{{\rm{C}}}^{\left(1\right)}$. The structure can mimic either a photonic modulator^43^ when *w*_C_ ≳ *w*_BTO_ or a plasmonic modulator^49^ when *w*_C_ = 0. The refractive-index change Δ*n*_BTO_ as a function of frequency in this structure is given by equation (6), which is derived in Supplementary Note 4:

6 $$\Delta {n}_{{\rm{BTO}}}\left(f\right)=\eta \frac{\left(1+{R}_{w}\right)\left({\varepsilon }_{{\rm{BTO}}}\left(f\right)-1\right)}{1+{R}_{w}\frac{{\varepsilon }_{{\rm{BTO}}}\left(f\right)}{{{{\varepsilon }}}_{{\rm{C}}}}}.$$

Here *R*_*w*_ = 2*w*_C_/*w*_BTO_ is the ratio of the total cladding width to BTO width and the factor *η* is a group of constant terms that are irrelevant to the present analysis. To arrive at equation (6), we exploited the boundary condition *ε*_C_*E*_C_ = *ε*_BTO_*E*_BTO_ for electric fields that are perpendicular to the cladding–BTO interface (Fig. 5a, blue line). If *ε*_BTO_ decreases, then *E*_BTO_ must increase to maintain continuity at the interface. As a result, a decreasing *ε*_BTO_ can counteract a decreasing *r*_BTO_ (equation (1)). We have also used Miller’s rule—which relates ${\chi }_{{\rm{BTO}}}^{\left(1\right)}$ and ${\chi }_{{\rm{BTO}}}^{\left(2\right)}$—to eliminate any dependence on ${\chi }_{{\rm{BTO}}}^{\left(2\right)}$ or *r*_BTO_.

There are two interesting cases of *R*_*w*_ corresponding to plasmonic and photonic devices. First, in the plasmonic devices in which *w*_C_ and *R*_*w*_ are zero, equation (6) simplifies to

7 $${\Delta n}_{{\rm{BTO}}}\left({R}_{w}=0\right)=\eta \left({\varepsilon }_{{\rm{BTO}}}-1\right).$$

This implies that any frequency dependence of *ε*_BTO_ will also be apparent in Δ*n*_BTO_. Figure 5b shows the frequency dependence of Δ*n*_BTO_ for various *R*_*w*_ values and with *ε*_C_ = 4. For *R*_*w*_ = 0 (dashed line), the shape of the curve closely follows the frequency response of *ε*_BTO_ (green) that was measured in this work. Second, typical photonic devices will have *R*_*w*_ ≈ 1 or larger. For the case of large *R*_*w*_, equation (6) can be approximated as

8 $${\Delta n}_{{\rm{BTO}}}\left({R}_{w}\gg 1\right)\approx \eta {\varepsilon }_{{\rm{C}}}\frac{{\varepsilon }_{{\rm{BTO}}}-1}{{\varepsilon }_{{\rm{BTO}}}},$$

where the last term is approximately unity when *ε*_BTO_ is large. The implication of this cannot be understated; Δ*n*_BTO_ essentially does not depend on *ε*_BTO_ or *r*_BTO_ when *ε*_BTO_ is sufficiently large. Figure 5b shows that this is true even for *R*_*w*_ = 1 (solid line) where Δ*n* is constant.

We note that the focus here is on the frequency response, which is why Δ*n*_BTO_ is plotted relative to its low-frequency value (Fig. 5b). Small values of *R*_*w*_ still give large values of Δ*n*_BTO_ in absolute terms, which is expected from plasmonic devices. However, this analysis highlights a trade-off for BTO devices between the highly efficient response of plasmonics and the frequency-independent response of photonics. Nevertheless, it might explain why previous demonstrations of BTO plasmonic modulators show a frequency-dependent EO response, whereas the photonic counterparts do not^43,49,52^.

## Conclusion

The frequency response of the permittivity and Pockels coefficients of LN and BTO have been measured. The data cover a broad frequency range important to a variety of developing technologies including high-speed communications, quantum networks, programmable photonics and reconfigurable metasurfaces. It provides a foundation for the design of novel devices operating up to the highest frequencies. The phase shifter and measurement method provide an avenue for exploring the EO properties of new materials as they are developed. Finally, the insights into the relationships between the refractive-index change, the BTO permittivity and the geometry of the device in which it is used may spark innovative ideas for overcoming the frequency dependence of EO properties.

## Methods

### Device fabrication

The phase shifters were fabricated on BTO-on-insulator and LN-on-insulator substrates with a 485-nm BTO layer or a 300-nm LN layer and a 2-µm buried oxide layer. Access waveguides were patterned with electron-beam lithography and transferred onto the BTO/LN layers with inductively coupled plasma reactive ion etching. The samples were annealed in an O_2_ atmosphere at 550 °C for 1 h to reduce optical propagation losses^42^. The 100-nm SiO_2_ spacer layer was deposited using plasma-enhanced chemical vapour deposition. Next, the grating couplers were fabricated from 130 nm of amorphous silicon that was deposited using plasma-enhanced chemical vapour deposition, patterned with electron-beam lithography and etched with inductively coupled plasma reactive ion etching using HBr plasma. The electrodes were deposited using a lift-off process in which electron-beam lithography was used for patterning and electron-beam evaporation was used to deposit 200 nm of gold. In the final step, a polymethyl methacrylate cladding was spin-coated onto the sample.

This simple process has multiple benefits. The dimensions of the structure are well defined and easy to reproduce across fabrication runs. The layer thicknesses are precisely controlled by modern deposition techniques and can be confirmed through ellipsometry. In Supplementary Note 5, simulations are used to show that uncertainties in layer thickness only have a small effect on the phase shifter’s voltage–length product. The only lithographic patterning required in the phase-shifter section is for the electrodes. There is no critical alignment with other lithography layers, and the micrometre-sized gap between the electrodes is easily achieved using photolithography. Additionally, there is no etching required in the phase-shifter section. This means that there is little uncertainty with respect to etching depths, waveguide widths or sidewall angles. Furthermore, there is no etching by-product or physical damage introduced into the film that may alter the EO properties.

### EO measurement setup

The setup used for EO measurements is described here. A tunable laser source provides the optical carrier, which passes through a polarization controller before being coupled to the chip with grating couplers. Waveguides route the optical signal to the phase modulators, which are driven by an external source (*f*_RF_) through the RF probes. The phase shifters are driven as an open load at the end of the probe without a termination because they have a length of only 25 µm for BTO or 50 µm for LN. All the measurements were performed without a d.c. bias but after the poling process (Supplementary Note 3). The modulated optical signal is coupled out from the chip with another grating coupler, and its spectrum is recorded using an optical spectrum analyser. Four different sources were used to generate the modulating signals across the full frequency range. An analogue signal generator (Agilent E8257D) was used to generate signals up to 70 GHz. The same signal generator was used to drive various frequency multipliers to generate frequencies above 70 GHz. The range between 70 GHz and 110 GHz was generated using a frequency multiplier (Radiometer Physics AFM6 75-110 + 10). Vector network analyser (VNA) extension modules (VDI VNAX WR6.5 and WR3.4) were used as frequency multipliers for the ranges of 110–170 GHz and 220–330 GHz.

The VNA measurements were performed using a Keysight PNA-X N5247B device in the one-port configuration and using the various VNAX extension modules from VDI to reach up to 330 GHz.

### Extracting permittivity from VNA measurements

Our method for measuring permittivity is based on *S*_11_ reflection measurements performed using a VNA. The *S*_11_ coefficient is equivalent to the reflection coefficient and is determined as *S*_11_ = (*Z* – *Z*_0_)/(*Z* + *Z*_0_), where *Z* is the phase-shifter impedance and *Z*_0_ = 50 Ω is the reference impedance of the system. The total impedance of a phase shifter can, therefore, be determined from

9 $$Z={Z}_{0}\times \frac{1+{S}_{11}}{1-{S}_{11}}.$$

The measured *Z* is then compared with a theoretically calculated impedance *Z*_theory_(*f*, *ε*_EO_) of an equivalent circuit model for the phase shifter under test, where *f* is the frequency and *ε*_EO_ is the complex permittivity of the EO layer. The measured permittivity is determined by finding the *ε*_EO_ value that makes the best match between *Z*_theory_ and the measured *Z*. More details on the equivalent circuit and the calculation of its impedance elements are provided in Supplementary Note 2. To validate our model, we use the average values of LN permittivity from the literature. The model primarily depends on the in-plane permittivity; therefore, *ε*_EO_ = *ε*_C_ = 27 is inserted into the calculation of *Z*_theory_(*f*, *ε*_EO_). The model gives an excellent fit to the measured impedance (Supplementary Note 2). The BTO impedance is not effectively fit for any constant value of *ε*_EO_. Instead, we use equation (2) to calculate *ε*_EO_, and the impedance model *Z*_theory_(*f*, *ε*_EO_(*γ*_0_, *σ*, *S*_R_, *ε*_∞_)) is dependent on the same parameters as the Debye permittivity model of equation (2). The complex permittivity given by the Debye model is essential to the impedance model for the purpose of calculating the dielectric loss. The results for the measured and fitted impedance of BTO are provided in Supplementary Note 2.

### EO simulations

This section describes the procedure for calculating the theoretical values of the voltage–length product *V*_π_*L*. The half-wave voltage *V*_π_ is defined as the voltage required to change the phase of an optical signal by π. If Δ*β* = *β*|_1V_ – *β*|_0V_ is the difference between the propagation constant of an optical mode with and without an applied voltage, then the total phase difference after propagation through a modulator with length *L* is

10 $$\Delta \varphi =\Delta \beta L=\frac{2\uppi L}{\lambda }\Delta {n}_{{\rm{eff}}}=\frac{2\uppi L}{\lambda }({\left.{n}_{{\rm{eff}}}\right|}_{1{\rm{V}}}-{\left.{n}_{{\rm{eff}}}\right|}_{0{\rm{V}}}).$$

To calculate the effective indices *n*_eff_|_1V_ and *n*_eff_|_0V_, EO simulations were performed using Lumerical’s MODE and DEVICE simulation software (version 2022 R1). In the first step, electrical simulations were performed in DEVICE to calculate the RF electric-field profile when a 1 V potential difference is applied to the electrodes. A small-signal a.c. perturbation was used to calculate the field profile at different RF frequencies. Electrical simulations were performed for various permittivities of the LN/BTO layers. Next, the simulated RF electric field was used with the full Pockels tensor for each material to calculate the spatially varying refractive-index profile in the LN/BTO layer. Values for the Pockels coefficients were taken from the literature^25,53^. Optical simulations were performed in MODE to find the effective index *n*_eff_ of the mode supported by the phase shifter. Two simulations were performed: one without the external RF field to get *n*_eff_|_0V_, and one with the external RF field to get *n*_eff_|_1V_. To achieve a phase shift Δ*φ* = π with the Δ*n*_eff_ calculated from an applied voltage of 1 V, the phase shifter must have a length *L*_π_ equal to

11 $${L}_{\uppi }=\left(\Delta \varphi =\uppi \right)\frac{\lambda }{2\uppi \Delta {n}_{{\rm{eff}}}}=\frac{1}{2}\frac{\lambda }{\Delta {n}_{{\rm{eff}}}}.$$

As implied by its name, the voltage–length product is the product of the applied voltage and the phase-shifter length. Because the applied voltage in this case is 1 V, the voltage–length product is then *V*_π_*L* = *VL*_π_ = (1 V) × *L*_π_ = *L*_π_. The simulated voltage–length product is, therefore, given by equation (11), which is restated as

12 $${V}_{\uppi }L=\frac{1}{2}\frac{\lambda }{\Delta {n}_{{\rm{eff}}}}.$$

### Extracting *r*_eff_ from phase modulation measurements

EO modulators are typically used to change the phase of an optical mode using an electrical drive signal. Such phase changes are also accompanied by changes to the optical spectrum. New frequencies are generated at *ω*_±*k*_ = *ω*_0_ ± *kω*_RF_, where *ω*_0_ is the optical carrier frequency, *ω*_RF_ is the RF modulation frequency and *k* = ±1, ±2… is an integer. The intensities of each frequency in the optical spectrum at the output of the modulator are given by Bessel functions and depend on the modulation efficiency^54^. Crucially, this allows the EO strength to be extracted from optical intensity measurements rather than phase-change measurements, which can be troublesome, especially at high frequencies.

The modulation efficiency, or alternatively, the half-wave voltage *V*_π_, of a phase shifter can be calculated from the intensity ratio between the optical carrier and the first modulation sideband as^54^

13 $${V}_{\uppi }={V}_{{\rm{RF}}}\frac{\uppi }{2}\sqrt{\frac{I\left({\omega }_{0}\right)}{I\left({\omega }_{0}\pm {\omega }_{{\rm{RF}}}\right)}},$$

where *I*(*ω*_0_) is the intensity of the optical carrier, *I*(*ω*_0_ ± *ω*_RF_) is the intensity of the first modulation sideband and *V*_RF_ is the peak voltage of the electrical modulation signal across the phase-shifter electrodes. The two intensities can be measured using an optical spectrum analyser; thus, if one knows the *V*_RF_, then *V*_π_ can be easily calculated.

Determining *V*_RF_, however, is not always trivial because it depends on the impedance and reflection characteristics of the phase shifter. Conveniently, the frequency-dependent reflection is already available from the electrical *S*_11_ measurements from which the permittivity was extracted. The phase shifter can be conceptualized as a termination at the end of an RF transmission line that delivers the driving signal. In this case, the voltage across the electrodes of the phase shifter is equal to *V*_RF_ = *V*_drive_(1 + *S*_11_). In other words, *V*_RF_ is the sum of the incoming and reflected electrical waves having amplitudes *V*_drive_ and *S*_11_ × *V*_drive_, respectively. *V*_RF_ can, therefore, be determined with high accuracy by combining the measurements of *S*_11_ along with a calibration of the RF losses between the RF source and phase shifter.

Next, an expression is needed to relate *V*_π_ to *r*_eff_. We start from the basis of equations (1) and (10). To relate the two equations requires an extra term that describes how much the effective index of the optical mode will change (Δ*n*_eff_) for a given refractive-index change of the EO material (Δ*n*_EO_). This term is known as the field interactor factor *Γ* (ref. ^15^):

14 $$\varGamma =\frac{\Delta {n}_{{\rm{eff}}}}{\Delta {n}_{{\rm{EO}}}}.$$

*Γ* accounts for the spatial variation in the RF and optical fields, as well as the overlap between the two fields and the active material (that is, LN or BTO). Inserting Δ*n*_eff_ = Δ*n*_EO_*Γ* into equation (10) and then using equation (1) to replace Δ*n*_EO_ gives

15 $$\Delta \varphi =\frac{\uppi L}{\lambda }{n}_{{\rm{EO}}}^{3}{r}_{{\rm{eff}}}\varGamma \frac{V}{{w}_{{\rm{gap}}}},$$

where the electric field *E* in equation (1) has been replaced by *V*/*w*_gap_, with *w*_gap_ being the width of the gap between the electrodes. Once again, setting Δ*φ* = π as per the definition of *V*_π_ gives an expression for the voltage–length product as

16 $${V}_{\uppi }L=\frac{\lambda {w}_{{\rm{gap}}}}{{n}_{{\rm{EO}}}^{3}{r}_{{\rm{eff}}}\varGamma }.$$

The effective Pockels coefficient *r*_eff_ can then be calculated from the measured *V*_π_ after rearranging equation (16) to be

17 $${r}_{{\rm{eff}}}=\frac{\lambda {w}_{{\rm{gap}}}}{{n}_{{\rm{EO}}}^{3}\varGamma {V}_{\uppi }L}.$$

### Distribution of ferroelectric domains in BTO

This section describes in further detail the framework for describing *r*_eff_ in a film with multiple ferroelectric domains. First, consider the case in which the thin film consists entirely of a single domain (*φ* = 0°, *ν*_*φ*=0°_ = 1) as is the case for LN. For materials like LN in which the *r*_33_ = *r*_*zzz*_ Pockels coefficient is the largest, *r*_eff_ as calculated using equation (4) has a maximum for *θ* = 90° when the optical and RF fields are polarized along the *c* axis (that is, the *z* axis). Figure 3b shows the dependence of *r*_eff_ on *θ* for LN using values^25^ of *r*_33_ = 31 pm V^–1^, *r*_42_ = 18 pm V^–1^ and *r*_13_ = 9 pm V^–1^. By contrast, BTO’s largest Pockels coefficient is *r*_42_, so it reaches a maximum *r*_eff_ for some angle *θ* that is not parallel to one of the crystal’s primary axes. This happens because *r*_42_ = *r*_*yxy*_ requires the **E**_Optical_ and **E**_RF_ components along two orthogonal crystal axes. The phase shifters in this work, however, have **E**_Optical_ and **E**_RF_ aligned in the same direction. The device and electric fields must, therefore, be rotated by an angle *θ* such that there will be projections of the fields onto the two orthogonal crystal axes. The dependence of *r*_eff_ versus *θ* for a hypothetical single-domain BTO thin film is shown in Fig. 3c (solid curve). The maximum *r*_eff_ value in this case is for waveguides aligned with *θ* ≈ 36°. For BTO, we used values^50^ of *r*_33_ = 30 pm V^–1^, *r*_42_ = 560 pm V^–1^ and *r*_13_ = 6 pm V^–1^. Both LN and BTO used in the analytical calculations are the clamped Pockels coefficients measured at frequencies near 10 MHz.

BTO thin films with an in-plane *c* axis typically consist of multiple domains oriented at right angles to each other. If the domains are randomly distributed and all the orientations of *φ* are equally likely (*ν*_*φ*=0°,90°,180°,270°_ = 0.25), then the Pockels effect from neighbouring domains will cancel out on average and *r*_eff_ is zero for all *θ* (Fig. 3c, dotted line). A non-zero *r*_eff_ requires an unequal distribution of domain orientations. This can be achieved through the application of a d.c. bias field between the two electrodes, which can flip the ferroelectric polarization in a domain by 180°. For example, a bias applied between the ground and signal electrodes (Fig. 3a) could flip the *φ* = 180° domain to a *φ* = 0° domain and the *φ* = 270° domain to a *φ* = 90° domain. This effect is illustrated in Fig. 3d, where the arrows represent the polarization direction within each domain. In a multidomain BTO film, the ideal distribution of domains should have no antiparallel domains to maximize *r*_eff_. This means that the relative domain fractions for a fully poled film should be *ν*_*φ*=0°,90°_ = 0.5 and *ν*_*φ*=180°,270°_ = 0, or vice versa. Figure 3c (dashed line) shows that *r*_eff_ in this situation is the maximum when *θ* = 45° and it is slightly less than the maximum for a single-domain film.

A more realistic case for our experiments consists of a majority of the domains polarized in a preferred direction, but with some antiparallel domains still present. For this partially poled case, we use domain fractions of *ν*_*φ*=0°,90°_ = 0.375 and *ν*_*φ*=180°,270°_ = 0.125. These values were reported for similar thin films without the presence of an external bias field but after applying an initial poling step to set the majority of the domains in the correct orientation^43^. The *r*_eff_ value for this case is plotted in Fig. 3c (dash–dotted line) and is also the maximum for *θ* = 45°. In fact, the curve for this partially poled case is the same as that for the fully poled case but scaled by a constant. This is a result of the counteracting contributions from the antiparallel domains to the overall EO effect.

### Relating *χ*^(1)^ and *χ*^(2)^ through Miller’s rule

The measured permittivity values give an insight into the frequency response of the Pockels coefficients through Miller’s rule, which states that there is a nearly constant ratio *δ* between the second-order susceptibility *χ*^(2)^(*ω*_1_, *ω*_2_, *ω*_3_) that enables a nonlinear process and the product of the first-order susceptibilities *χ*^(1)^(*ω*_1_), *χ*^(1)^(*ω*_2_) and *χ*^(1)^(*ω*_3_) corresponding to each of the frequencies involved in the process^47,51^. The Pockels effect in an optical phase shifter is essentially a sum/difference frequency process with an optical frequency *ω*_0_, a modulating frequency *ω*_m_ and sum/difference frequencies *ω*_0_ ± *ω*_m_. In the form of Miller’s rule, this looks like equation (18).

18 $$\delta =\frac{{\chi }^{\left(2\right)}\left({\omega }_{0}\pm {\omega }_{{\rm{m}}},\,{\omega }_{0},{\omega }_{{\rm{m}}}\right)}{{\chi }^{\left(1\right)}\left({{{\omega }}}_{0}\pm {{{\omega }}}_{{\rm{m}}}\right){\chi }^{\left(1\right)}\left({{{\omega }}}_{0}\right){\chi }^{\left(1\right)}\left({{{\omega }}}_{{\rm{m}}}\right)}$$

For the case of the measurements presented here, the optical carrier has a fixed frequency *ω*_m_ ≈ 193 THz (*λ* = 1,550 nm), and therefore, *χ*^(1)^(*ω*_0_) is constant. Additionally, *ω*_0_ is much larger than the modulating frequencies between 100 MHz and 330 GHz. This means that *ω*_0_ ± *ω*_m_ ≈ *ω*_0_ for all *ω*_m_, and therefore, *χ*^(1)^(*ω*_0_ ± *ω*_m_) is also constant. If *δ*, *χ*^(1)^(*ω*_0_) and *χ*^(1)^(*ω*_0_ ± *ω*_m_) are all constant, then any change in the value of *χ*^(1)^(*ω*_m_) must also be accompanied by a similarly proportioned change in *χ*^(2)^(*ω*_0_ ± *ω*_m_, *ω*_0_, *ω*_m_) and vice versa. Because the dielectric constant can be expressed as *ε* = 1 + *χ*^(1)^ and the Pockels coefficient can be expressed as *r* = 2*χ*^(2)^/*n*^4^, it can, therefore, be inferred that when measurements show changes in the dielectric constant as a function of frequency, there are similar changes occurring in the Pockels coefficients. This also allows the Pockels coefficient to be expressed as a product of the first-order susceptibilities:

19 $$r\approx \frac{2\delta }{{n}_{0}^{4}}{\left({\chi }^{\left(1\right)}\left({{{\omega }}}_{0}\right)\right)}^{2}{\chi }^{\left(1\right)}\left({{{\omega }}}_{{\rm{m}}}\right).$$

This can also be expressed in terms of the optical refractive index and RF permittivity by considering that *n*^2^ = *ε* = 1 + *χ*^(1)^. The form of equation (5) is reached after making the relevant substitutions for the optical and RF susceptibilities:

20 $$r\approx 2\delta \frac{{\left({n}_{0}^{2}-1\right)}^{2}}{{n}^{4}}\left(\varepsilon -1\right).$$

## Online content

Any methods, additional references, Nature Portfolio reporting summaries, source data, extended data, supplementary information, acknowledgements, peer review information; details of author contributions and competing interests; and statements of data and code availability are available at 10.1038/s41563-025-02158-1.

## Supplementary information

Supplementary Information Supplementary Notes 1–5.

## Data Availability

The data that support this work are presented in the article and its Supplementary Information. Further data are available from the corresponding authors upon reasonable request.
