# Supplementary material for: Barium titanate and lithium niobate permittivity and Pockels coefficients from megahertz to sub-terahertz frequencies
Source: Nat Mater. 2025 Mar 17;24(6):868–75. doi: 10.1038/s41563-025-02158-1 (PMC12133585; doi:10.1038/s41563-025-02158-1)
Supplement: Supplementary file 1 — Supplementary Notes 1–5. [file 41563_2025_2158_MOESM1_ESM.pdf]

nature materials

Supplementary information

https://doi.org/10.1038/s41563-025-02158-1

Barium titanate and lithium niobate permittivity and Pockels coefficients from megahertz to sub-terahertz frequencies

In the format provided by the authors and unedited

Table of Contents

1 Origins of the Frequency Dependence of the Permittivity and Pockels Coefficients 2
1.1 Factors Influencing the Frequency Dependence of the Permittivity in BTO ... 2
2 Extracting Permittivity from S_{11} Reflection Measurements 3
2.1 Impedance Measurements and Models 4
2.2 Equivalent Circuit Model of the Phase Shifter 6
2.3 Calculation of the Circuit Elements 7
2.3.1 $C\mathrm{GSG}$ and $C\mathrm{si}$ – Coplanar Waveguide Capacitance 7
2.3.2 $G\mathrm{EO}$ – Dielectric Loss in the EO Layer 9
2.3.3 $G\mathrm{si}$ – Silicon Conductance 10
2.3.4 $C\mathrm{sub}$ – Signal-Substrate Capacitance 10
2.3.5 Longitudinal Impedance 10
3 Extracting Pockels Coefficients From Phase Modulation Measurements 12
3.1 Derivation of the Angular Dependence of $r\mathrm{eff}$ 12
3.1.1 Angular Dependence of the Permittivity 14
3.2 Fitting $r\mathrm{ij}$ Coefficients to the Measured $r\mathrm{eff}$ Data 14
3.3 Poling Dynamics 15
4 Derivation of the Dependence of Δn on $\varepsilon\mathrm{BTO}$ 17
5 Voltage-Length Product Dependencies 19
5.1 Varying Ferroelectric Permittivity 19
5.2 Varying Layer Thicknesses 19
References 21

1 Origins of the Frequency Dependence of the Permittivity and Pockels Coefficients

The permittivity and Pockels coefficients are frequency dependent because they originate from piezoelectric, ionic and electronic contributions that die out above their respective excitation frequencies[1,2]. At frequencies from DC to ~1 MHz there are contributions from the mechanical deformation of a lattice due to the piezoelectric effect and from lattice vibrations related to acoustic phonons. A distinction is typically made between the frequency regimes above and below the acoustic phonon frequencies which are typically between 1 and 100 MHz. Below 1 MHz a crystal is said to be free because it is free to deform under the influence of an external electric field. Above ~100 MHz a crystal is said to be clamped because these mechanical deformations no longer occur due to their slow timescale. Nonlinearities related to optical phonons also contribute to the Pockels effect. This is commonly referred to as the ionic contribution and it dies out at optical phonon frequencies which are usually around a few THz. Finally, the highest frequency contributions to EO effects originate from electronic resonances which have characteristic frequencies in the PHz regime. A result of the various contributions is that different materials may have different EO responses in certain frequency ranges. Organic polymers are an example of materials that derive their nonlinearity almost exclusively from electrons[2]. They maintain strong EO effects up to optical frequencies and have been demonstrated in photonic integrated circuits with phase shifters operating up to at least 500 GHz[3] and in antennas operating up to 2.4 THz[4]. However, inorganic materials are often preferred for their temperature stability and compatibility with common fabrication processes.

The primary contribution to the nonlinearities in ferroelectric crystals comes from ionic resonances. One consequence of the EO strength being dominated by acoustic and optical lattice vibrations is that their contributions can be very different between materials. For example, the largest Pockels coefficient in BTO[5] is more than an order of magnitude larger than that for LN[6], yet the two materials still have similar optical properties which are determined from electronic resonances. Furthermore, the EO strength can be dispersive since the acoustic lattice vibrations dissipate between MHz and GHz frequencies, which is the range of interest to many of the applications mentioned in the introduction of the main text.

1.1 Factors Influencing the Frequency Dependence of the Permittivity in BTO

We attribute defects, domain structure and domain poling to be the main reasons behind the variation among the literature data in terms of the strength and the central component of the low frequency relaxation. The role of defects has been covered in the main text. Here, we examine the differences between single and poly domain crystals. Then we look the domain size and the effects of antiparallel and 90-degree ferroelectric polarizations (i.e. poling).

The difference between single and poly domain BTO is straightforward. In a single domain crystal, the pure *a*- or *c*-axis permittivity can be measured whereas in poly domain

BTO the permittivity will always be a mix of *a*- and *c*-axis domains. Indeed, a previous study prepared BTO samples with varying fractions of 90-degree domains and measured the permittivity along the supposed *c*-axis[7]. They found that the permittivity increased in samples with more 90-degree domains than in those with only 180-degree domains because the 90-degree domains would contribute ε_a rather than ε_c to the effective permittivity. The opposite must then be true for measurements along the *a*-axis, where the presence of 90-degree domains reduces the permittivity due to an increased influence from ε_c. This might explain why Ref. 31 in Fig. 2d – the only one with a single domain sample that can measure pure ε_a – starts with a rather high permittivity at low frequencies and then has a large drop.

As an example of the differences between poly domain crystals, consider the following. An *a*-axis BTO thin film like ours can have multiple domains where the *c*-axis is randomly oriented in one of four 90-degree orientations in the plane of the film. Along one of these 90-degree directions, half of the domains will be *c*-axis and the other half will be *a*-axis on average. In Supplementary Note 3, we show that in this case the effective permittivity should be an equal-weighted average of ε_a and ε_c. Compare this to Refs. 33 and 35 of the main text which report data on a thin film and a ceramic, respectively. Both samples feature a mixture of domains where the *c*-axis is randomly oriented in all directions (i.e. not just in-plane). Now the effective permittivity is skewed towards ε_a because the unit cells have two *a*-axes but only one *c*-axis. Since $\varepsilon_a \gg \varepsilon_c$, the permittivity will be larger in mixed *c*- and *a*-axis samples compared to an *a*-axis thin film like ours.

The variation in the relaxation frequency in the MHz-GHz is correlated with the domain size. Higher relaxation frequencies are associated with smaller domains (3 GHz for 230 nm vs. 770 MHz for 980 nm)[8]. This may also explain why thin films tend to have relaxations in the GHz range while 100s of MHz is more common for bulk crystals with larger dimensions and larger domains.

Finally, we discuss the poling of thin films. Neighbouring domains can have ferroelectric polarizations that are parallel, antiparallel or at right angles to one another. Clemens et al. also found that the permittivity drop along the c-axis tends to be greater when there are more 90-degree domains[7]. By the same logic as before, the drop along the a-axis should be smaller with more 90-degree domains. Antiparallel domains seem to have an even more significant influence[9]. Nakao et al. prepared BTO crystals with only 180-degree domains and then poled the crystals in stages to gradually remove the antiparallel domains. They found that the as-prepared crystals with random domain polarizations had a factor 3 higher ε_c and a factor 2 higher ε_a when compared to fully poled films that had no antiparallel domains. In addition, the raw films with many antiparallel domains showed almost no frequency dependence while the fully poled films showed a large permittivity drop around 1 MHz.

2 Extracting Permittivity from S_{11} Reflection Measurements

The permittivity is found by fitting the impedance of an equivalent circuit model to the impedance measured with S_{11} reflection measurements. The methods section of the main text describes the fitting process.

Presented here are the measured impedance and S_{11} data as well as the equivalent circuit model that is used to analytically derive impedance and S_{11}. Using the circuit model one can determine the permittivity of the EO layer $\varepsilon_{\mathrm{EO}}$ from a measurement of S_{11}. This is

because the only free parameter of the impedance model is $\varepsilon_{\mathrm{EO}}$. All other parameters that go into the model are fixed because they are related to either the geometry of the structure or the permittivity and conductivity of other materials in the structure.

We use S_{11} reflection measurements because our phase shifters are small enough to effectively be a lumped element. Note that similar methods can be used for transmission lines if the phase shifters are longer[10,11].

2.1 Impedance Measurements and Models

To validate the impedance model, we use LN as a reference material. Literature data for LN's permittivity from many sources are in good agreement on the values of ε_c = 27 and ε_a = 45 (see Fig. 2(c) in the main text). Additionally, the literature agrees that these values should be constant over the measured frequency range. LN is a relatively simple case with few unknowns which makes it a good test case for the model.

The measured data for the real and imaginary parts of both Z and S_{11} are shown in Fig. S1 by dark blue circles. The gap in the data between 170 GHz and 220 GHz is a range that cannot be covered by our equipment. We then compare the measurements against Z and S_{11} values that are calculated from the equivalent circuit model (light blue lines). Towards this end we have used the values of ε_c and ε_a from literature and all other device geometry parameters were taken from the designed dimensions of the structure or commonly used material properties ($\varepsilon_{\mathrm{si}}$ = 11.7, $\varepsilon_{\mathrm{oxide}}$ = 3.9, $\varepsilon_{\mathrm{PMMA}}$ = 3.9, σ_{Au} = 4×10^8 S/m). The circuit model, its parameters and details on how to calculate its elements are described in the next section. None of the model parameters were fitted to match the measure data. The excellent match between the model and the measured data suggests that the impedance model is well-founded. The only part of the measurements that is not a near-exact match is the real part of Z at frequencies below 1 GHz. We presume it is the result of some dielectric loss that originates from dielectric resonances in the 1-100 MHz range[12,13].

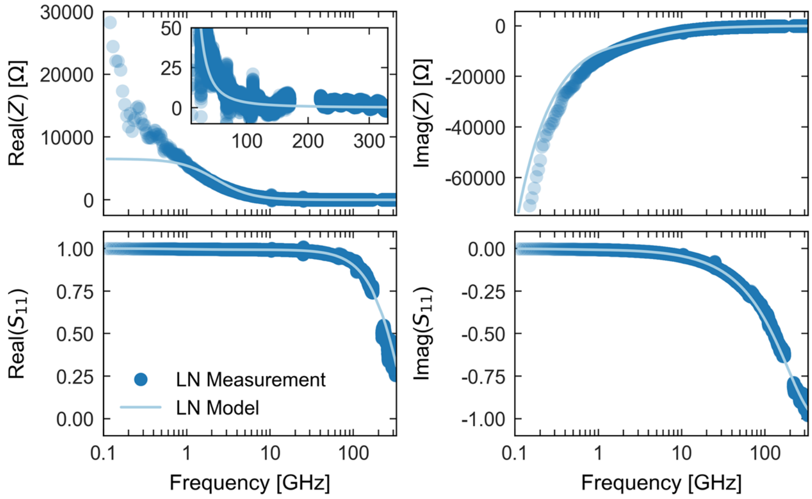

Fig. S1 | Measured and modelled impedance data for LN. The top row shows the real and imaginary parts of the impedance Z. The bottom row shows the real and imaginary parts of the S_{11} reflection parameter.

The excellent match between the LN impedance measurements and the impedance calculated from the model using known parameters, prove that the impedance model is suitable for the structures in this work.

To add further confluence towards the model's accuracy, we use the same process for LN devices with three different lengths. The measured S_{11}data and the S_{11} predicted by the model are plotted in Fig. S2. For the longer devices at the highest frequencies, the lumped element assumption of the model starts to break down because the RF wavelength starts to approach the device length. This is why the phase shifters used for EO characterization are so short.

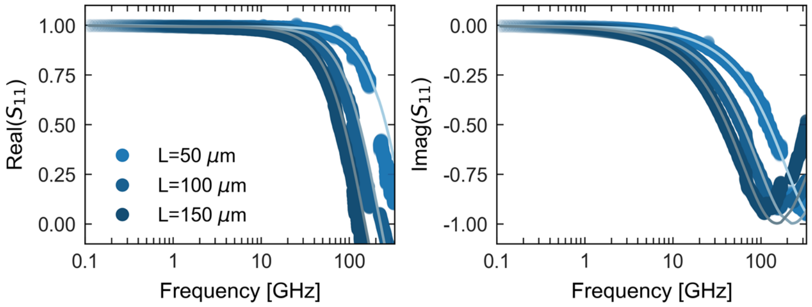

Fig. S2 | Measured and modelled reflection data for LN devices with different lengths. The real and imaginary parts of the S_{11} reflection parameter are shown for LN devices with lengths of 50 µm, 100 µm and 150 µm. The EO characterization is performed with the 50 µm devices to stay within the lumped element approximation at higher frequencies.

Now we focus on the application of the model to BTO devices. Modelling the BTO devices is more challenging because $\varepsilon_{\mathrm{BTO}}$ is expected to vary across the measured frequency range. Additionally, the dispersion of the real part of $\varepsilon_{\mathrm{BTO}}$ also must come with an imaginary component. There is also no widespread agreement about $\varepsilon_{\mathrm{BTO}}$ among previous reports as was the case for LN. Therefore, $\varepsilon_{\mathrm{BTO}}$ must be found by fitting an analytical model to the data. We use the same impedance model for BTO that was used for LN but with geometric parameters adapted for the BTO devices. Instead of the constant permittivity model assumed for LN, we use the Debye model in equation (2) of the main text to get a frequency dependent $\varepsilon_{\mathrm{BTO}}$. The Debye model takes four parameters as input: the central relaxation frequency γ_0, the standard deviation of relaxation frequencies in log-space σ and the relaxation strength S_{R}. The high frequency permittivity is taken to be ε_{∞} = 231 based on the Debye model fitted to literature data (Table 1, main text). These three unknown variables are now used as fitting parameters in the impedance model. Fig. **S3** shows the measured BTO data in the dark green circles. The results calculated from the circuit model best fitting values for the Debye model parameters are plotted in the light green lines. The parameters that give the best fit to the data along with the standard errors of the fit are given in Table 1 of the main text. The BTO impedance model describes the measurements remarkably well and the small standard errors of the fit give us a high degree of confidence that BTO's permittivity in this frequency range is well-described by the Debye model.

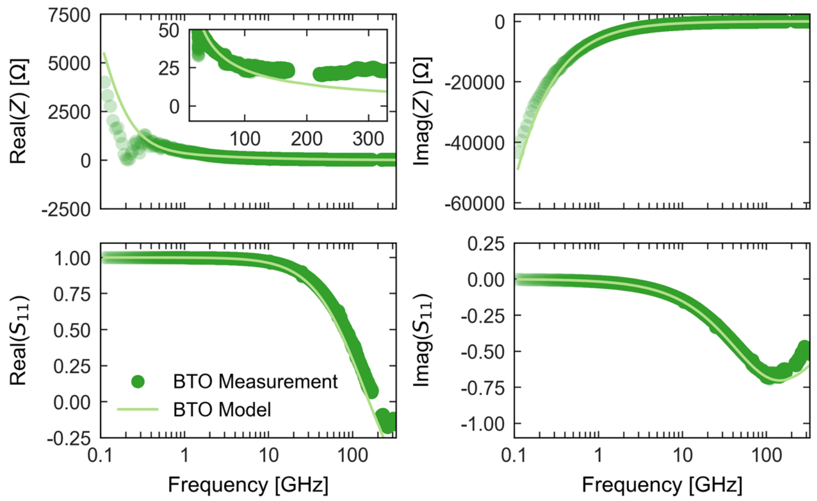

Fig. S3 | Measured and modelled impedance data for BTO. The top row shows the real and imaginary parts of the impedance Z. The bottom row shows the real and imaginary parts of the S_{11} reflection parameter.

2.2 Equivalent Circuit Model of the Phase Shifter

This section describes the equivalent circuit model that was used to model the impedance of the phase shifters and subsequently extract the permittivity of the thin films. The calculation of the elements in the equivalent circuit will be described in the next section.

Fig. S4(a) shows a cross-sectional schematic of the phase shifter's layer stack overlaid with the various elements that contribute to the overall impedance. Between the signal and ground electrodes there exists a capacitance C_{GSG} which is the sum of partial capacitances due to each layer in the stack. In parallel to C_{GSG} is the conductance in the electro-optic (EO) layer G_{EO}. The conductance represents the dielectric loss in the EO layer and for BTO it is essential for an accurate impedance model. Also in parallel to C_{GSG} and G_{EO} is the substrate impedance. The silicon substrate requires multiple elements to account for the conduction and displacement currents that depend on the modulation frequency[14,15]. At low frequencies substrate currents are dominated by carrier conduction and this is accounted for with G_{si}. Displacement currents dominate at high frequencies and this is accounted for with the capacitive element C_{si}. A second capacitor C_{sub} accounts for the capacitance between the signal electrode and the silicon substrate. Fig. S4(b) shows the arrangement of these elements in a simplified equivalent circuit model that highlights the parallel current paths. We note that here we show the full GSG electrode structure, in contrast to the figures of the main text where typically only one ground electrode is shown. The reason for this is that our high-frequency electrical probes have a GSG configuration. Fig. S4(c) shows how the phase shifters were incorporated into coplanar waveguide electrodes to be compatible with GSG probes. Each set of electrodes supports two phase shifters with one in each G-S gap, similar to Mach-Zehnder modulators. Unlike Mach-Zehnder modulators, however, each arm stays separate and has its own input and output grating couplers. This configuration has the advantage of doubling the number of phase shifters available to measure.

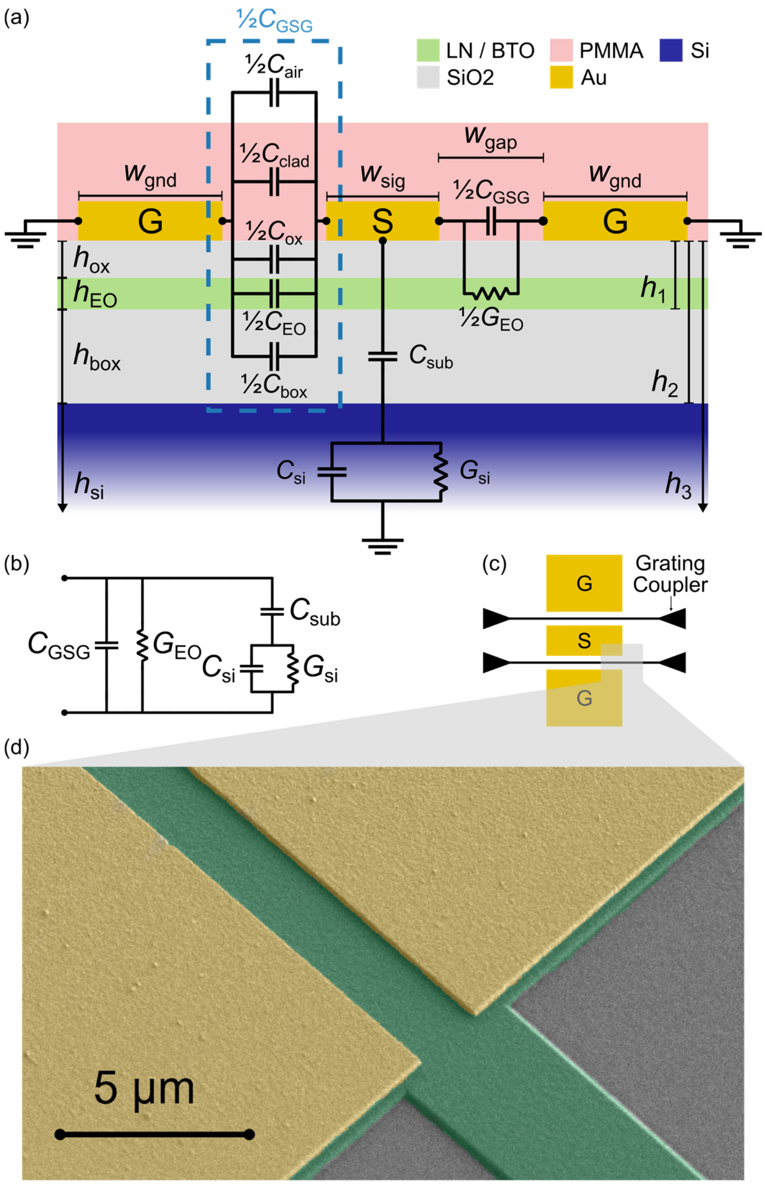

Fig. S4 | Equivalent circuit model of the phase shifter. **a**, Schematic of the various contributions to the total impedance of the phase shifter. **b**, Simplified Equivalent circuit model of the phase shifter. **c**, Illustration of the on-chip configuration of the phase shifters. Each GSG electrode supports two phase shifters, one in each G-S gap. This configuration is necessary to maintain compatibility with high-frequency electrical probes. **d**, Scanning electron microscope image of the transition from the waveguide to the start of the phase shifter.

2.3 Calculation of the Circuit Elements

This section describes the calculation of the individual circuit elements in Fig. S4.

2.3.1 $\boldsymbol{C_{\mathrm{GSG}}}$ and $\boldsymbol{C_{\mathrm{si}}}$ – Coplanar Waveguide Capacitance

The electrodes form a coplanar waveguide (CPW) which has a capacitance that can be calculated by conformal mapping methods[14,15]. For multi-layer substrates the capacitance of a CPW can be calculated with the following elliptic integrals[16]. The general form for the capacitance per unit length of a CPW with an effective permittivity $\varepsilon_{\mathrm{eff}}$ is given by

$$C_{\mathrm{CPW}} = 4\varepsilon_0\varepsilon_{\mathrm{eff}}\frac{\mathrm{K}(k_0')}{\mathrm{K}(k_0)}\ , \tag{1}$$

where K is the complete elliptic integral of the first kind with modulus k_0 given by equation (2) and $k_0' = \sqrt{1 - k_0^2}$

$$k_0 = \frac{x_\mathrm{c}}{x_\mathrm{b}} \sqrt{\frac{x_\mathrm{c}^2 - x_\mathrm{a}^2}{x_\mathrm{b}^2 - x_\mathrm{a}^2}}. \quad (2)$$

The parameters x_a, x_b, x_c are related to the CPW geometry

$$\begin{aligned} x_\mathrm{a} &= \frac{w_\mathrm{sig}}{2}, \\ x_\mathrm{b} &= x_\mathrm{a} + w_\mathrm{gap}, \\ x_\mathrm{c} &= x_\mathrm{b} + w_\mathrm{gnd}. \end{aligned} \quad (3)$$

If the CPW is embedded in a homogeneous medium then $\varepsilon_\mathrm{eff} = \varepsilon_\mathrm{r}$ where ε_r is the permittivity of the medium. For multi-layer structures the partial capacitance technique allows one to calculate the capacitance contribution for each material layer individually. The total capacitance is then be given by the sum of the parallel partial capacitances. The calculation of the partial capacitances is modified from equation (1). Taking the silicon substrate in Fig. S4(a) as an example, one would first calculate the partial capacitance for a silicon layer that spanned the entire thickness h_3 and then subtract from that the partial capacitance of a silicon layer that spanned the thickness h_2. The remaining capacitance is that of the actual silicon layer. The elliptic integrals take a different form[16]

$$C_\mathrm{si} = 2\varepsilon_0\varepsilon_\mathrm{si}\frac{\mathrm{K}(k_3')}{\mathrm{K}(k_3)} - 2\varepsilon_0\varepsilon_\mathrm{si}\frac{\mathrm{K}(k_2')}{\mathrm{K}(k_2)}, \quad (4)$$

with the moduli k_i now given by equation (5), $k_\mathrm{i}' = \sqrt{1 - k_\mathrm{i}^2}$ and i = 1, 2, 3 corresponding to the thicknesses h_i in Fig. S4(a)

$$k_\mathrm{i} = \frac{\sinh\left(\frac{\pi x_\mathrm{c}}{2h_\mathrm{i}}\right)}{\sinh\left(\frac{\pi x_\mathrm{b}}{2h_\mathrm{i}}\right)} \sqrt{\frac{\sinh^2\left(\frac{\pi x_\mathrm{c}}{2h_\mathrm{i}}\right) - \sinh^2\left(\frac{\pi x_\mathrm{a}}{2h_\mathrm{i}}\right)}{\sinh^2\left(\frac{\pi x_\mathrm{b}}{2h_\mathrm{i}}\right) - \sinh^2\left(\frac{\pi x_\mathrm{a}}{2h_\mathrm{i}}\right)}}. \quad (5)$$

The factor 2 in equation (4) compared to the factor 4 in equation (1) occurs because the calculation for the silicon (or any other layer) is only for the half-space below the CPW.

Equations (4) and (5) can become inaccurate when the permittivity for each layer is not strictly decreasing away from the electrodes[17,18]. This is especially true for the layer stacks in this work where the permittivities of LN and BTO are much larger than that of the silicon dioxide layer that separates the EO materials from the CPW. Modifications to the conformal mapping formulas above have been proposed[17,18], however, we found it easier and more accurate to use EM simulations to calculate the effective permittivity ε_eff of the CPW with the entire layer stack. This ε_eff can then be used with equation (1) to calculate the total CPW capacitance. The dependence of ε_eff on the permittivity of the EO layer ε_EO is shown in Fig. S5(a).

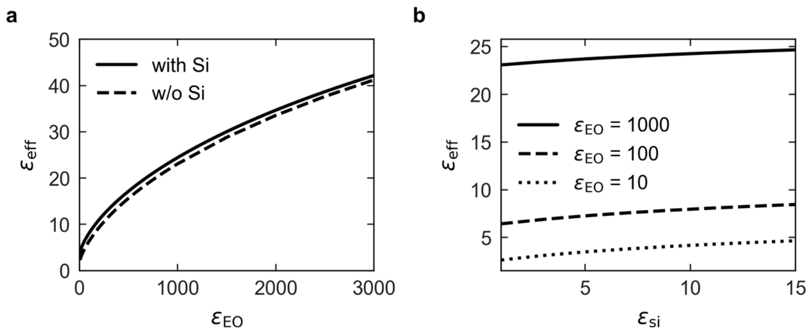

Fig. S5 | Effective permittivity of the phase shifter. a, CPW $\varepsilon_{\mathrm{eff}}$ dependence structure on the EO permittivity $\varepsilon_{\mathrm{EO}}$ with (solid) and without (dashed) the silicon substrate. **b**, CPW $\varepsilon_{\mathrm{eff}}$ dependence on $\varepsilon_{\mathrm{si}}$ for various values of $\varepsilon_{\mathrm{EO}}$ to verify that the presence of the silicon layer does not strongly influence the total field distribution and that the method to determine C_{si} is accurate.

Since C_{si} is in parallel with G_{si} it is still necessary to separate the capacitance due to the silicon layer from the capacitance due to the rest of the layer stack, which is labelled as C_{GSG}. To this end, we use a method similar to the idea of partial capacitances, where the capacitance of the full layer stack C_{CPW} should be the sum of parallel capacitances such that $C_{\mathrm{CPW}} = C_{\mathrm{GSG}} + C_{\mathrm{si}}$. First, the total capacitance C_{CPW} is calculated based on the $\varepsilon_{\mathrm{eff,\,CPW}}$ that is simulated with the full layer stack. Then $\varepsilon_{\mathrm{eff,\,GSG}}$ is simulated without the silicon layer to calculate C_{GSG}. Finally, C_{si} is given by the difference $C_{\mathrm{CPW}} - C_{\mathrm{GSG}}$. The effective permittivity with and without the silicon layer is shown in Fig. S5(a) by the solid and dashed lines, respectively. To verify that removing the silicon layer doesn't strongly influence the field distribution and thus, the value $\varepsilon_{\mathrm{eff,\,GSG}}$, C_{si} was calculated using various values for $\varepsilon_{\mathrm{si}}$ in the simulation. The capacitance should be linearly proportional to $\varepsilon_{\mathrm{si}}$ if the rest of the rest of the geometry is fixed. Fig. S5(b) shows that this is approximately true. Note that all capacitances in this section are per unit length, so the actual capacitance of the device is obtained by multiplying by the length of the CPW.

2.3.2 $\boldsymbol{G_{\mathrm{EO}}}$ – Dielectric Loss in the EO Layer

Dielectric loss occurs whenever a material's permittivity has an imaginary component. In general, when the real part of the permittivity changes there will also be an imaginary component as per the Kramers-Kronig relations. For LN this is not an issue in the measured frequency range. For BTO, the Debye model in equation (2) of the main text provides the complex permittivity $\varepsilon' + j\varepsilon''$ and the loss tangent can be calculated as $\tan\delta = \varepsilon''/\varepsilon'$. The dielectric loss in the BTO layer can be modelled as a conductance G_{EO} in parallel to C_{GSG}. Since the dielectric loss in the other layers is negligible compared to the loss in the BTO layer, the entire loss (or conductivity) must come from the active electro-optical structure, i.e. $G_{\mathrm{EO}} \approx G_{\mathrm{GSG}}$. Dielectric loss can be calculated with the same elliptic integrals as in the previous sections[19]. In fact, the dielectric loss is simply the capacitance multiplied by a factor $2\pi f \cdot \tan\delta$ which means that G_{EO} in the context of this analysis is given by

$$G_{\mathrm{EO}} = 2\pi f \cdot \tan\delta \cdot C_{\mathrm{GSG}} \ . \tag{6}$$

G_{EO} is essential to the impedance model if it is to match the real and imaginary parts of the measured impedance simultaneously. Anecdotally, if G_{EO} is excluded from the impedance model then $\varepsilon_{\mathrm{BTO}}$ will typically be overestimated, which would also lead to overestimated values for the Pockels coefficient. Note that equation (6) gives G_{EO} per unit

length and must therefore be multiplied by the length of the CPW to get the dielectric loss of an actual device.

2.3.3 $\boldsymbol{G_{\mathrm{si}}}$ – Silicon Conductance

The silicon conductance is calculated with conformal mapping in a similar way to its partial capacitance[15]

$$G_{\mathrm{si}} = \frac{2}{\rho_{\mathrm{si}}} \frac{\mathrm{K}(k_3')}{\mathrm{K}(k_3)} - \frac{2}{\rho_{\mathrm{si}}} \frac{\mathrm{K}(k_2')}{\mathrm{K}(k_2)}, \tag{7}$$

where ρ_{si} is the silicon resistivity and k_{i}, k_{i}' are given by equation (5). Once again, the conformal mapping method is not the most accurate for the layer stack in this work. However, G_{si} is so small for the high-resistivity silicon substrates in this work that G_{si} – and the inaccuracy of its calculation – is irrelevant to the impedance over the measured frequency range. If an accurate calculation of G_{si} is required, we propose the following method to calculate a correction term for the elliptic integrals. First, calculate the approximated silicon layer capacitance $C_{\mathrm{si,\,approx.}}$ according to equation (4). Then, calculate the actual silicon layer capacitance C_{si} using electromagnetic simulations as described in section 2.2.1. Finally, the ratio $\kappa = C_{\mathrm{si}}/C_{\mathrm{si,\,approx.}}$ gives a scaling factor that corresponds to how much the elliptic integrals under-/overestimate the actual quantity. Keeping the same notation where $G_{\mathrm{si,\,approx.}}$ is given by equation (7), then the actual silicon conductance would be given by $G_{\mathrm{si}} = \kappa G_{\mathrm{si,\,approx.}}$. Note that equation (7) gives G_{si} per unit length and must therefore be multiplied by the length of the CPW to get the silicon conductance of an actual device.

2.3.4 $\boldsymbol{C_{\mathrm{sub}}}$ – Signal-Substrate Capacitance

The signal-substrate capacitance is given by the standard parallel plate capacitance model

$$C_{\mathrm{pp}} = \varepsilon_0 \varepsilon_{\mathrm{r}} \frac{w_{\mathrm{sig}} l}{h}, \tag{8}$$

where l is the length of the CPW. The parallel plate capacitance is calculated for each of the layers between the signal electrode and the silicon substrate. The total signal-substrate capacitance is then

$$\begin{aligned} C_{\mathrm{sub}} &= \left(C_{\mathrm{sub,\,ox}}^{-1} + C_{\mathrm{sub,\,EO}}^{-1} + C_{\mathrm{sub,\,box}}^{-1}\right)^{-1} \\ &= \left(\left(\varepsilon_0 \varepsilon_{\mathrm{ox}} \frac{w_{\mathrm{sig}} L}{h_{\mathrm{ox}}}\right)^{-1} + \left(\varepsilon_0 \varepsilon_{\mathrm{EO}} \frac{w_{\mathrm{sig}} L}{h_{\mathrm{EO}}}\right)^{-1} + \left(\varepsilon_0 \varepsilon_{\mathrm{box}} \frac{w_{\mathrm{sig}} L}{h_{\mathrm{box}}}\right)^{-1}\right)^{-1}. \end{aligned} \tag{9}$$

Since these capacitances are in series, C_{sub} is dominated by the smallest capacitor which is the buried oxide layer. Since $h_{\mathrm{box}} \gg h_{\mathrm{EO}}$ and $\varepsilon_{\mathrm{box}} \ll \varepsilon_{\mathrm{EO}}$, there is a negligible dependence on $C_{\mathrm{sub,\,EO}}$ and therefore $\varepsilon_{\mathrm{EO}}$ as well.

2.3.5 Longitudinal Impedance

Most analyses of CPW impedance also include elements to account for longitudinal currents in the structure[15,19,20]. This includes the inductance and resistance of the electrodes, as well as the resistance of the silicon substrate in the propagation direction. These elements would be placed in series with the simplified equivalent circuit of Fig. **S4**(b). For the CPWs in this work, however, the device lengths are so small that the

longitudinal elements can be neglected for the measured frequency range. For completeness, details on the calculation of these elements are provided below.

The inductance of the electrodes L_{CPW} is given by conformal mapping with k_0 given by[14,20]

$$L_{\mathrm{CPW}} = \frac{4}{\mu_0} \frac{\mathrm{K}(k_0)}{\mathrm{K}(k_0')} , \tag{10}$$

where μ_0 is the permeability of free space. The resistance along the signal electrode R_{sig} depends on the skin depth δ of the electrode. The electrodes in our devices are only 200 nm thick and are much smaller than the skin depth except for frequencies above 300 GHz. Regardless, the skin depth can be calculated by[21]

$$\delta = \sqrt{\frac{2}{\sigma\omega\mu}} \sqrt{\sqrt{1 + \left(\frac{\omega\varepsilon}{\sigma}\right)^2} + \frac{\omega\varepsilon}{\sigma}} , \tag{11}$$

where ω is the angular frequency, σ is the material's conductivity, $\mu = \mu_0\mu_{\mathrm{r}}$ and $\varepsilon = \varepsilon_0\varepsilon_{\mathrm{r}}$. When the thickness of the electrodes $t \leq \delta$ then

$$R_{\mathrm{sig}} = \frac{1}{\sigma_{\mathrm{sig}} \cdot t \cdot w_{\mathrm{sig}}} , \tag{12}$$

where σ_{sig} is the conductivity of the electrode. When $t \geq \delta$ then R_{sig} depends on the skin depth – and therefore frequency as well – and is given by

$$R_{\mathrm{sig}} = \frac{1}{\sigma_{\mathrm{sig}} \cdot \delta_{\mathrm{sig}} \cdot w_{\mathrm{sig}}} . \tag{13}$$

Similarly, the longitudinal resistance in the silicon substrate is given by

$$R_{\mathrm{L}} = \frac{\rho_{\mathrm{si}}}{\delta_{\mathrm{si}} \cdot w_{\mathrm{sig}}} . \tag{14}$$

Where ρ_{si} is the resistivity of the silicon. L_{CPW} and R_{sig} are connected in series while R_{L} offers a parallel current path to the other two. Typically R_{L} is much greater than the impedance of R_{sig} and L_{CPW} and is therefore inconsequential.

3 Extracting Pockels Coefficients From Phase Modulation Measurements

3.1 Derivation of the Angular Dependence of r_{eff}

Here we derive the analytical formula for the effective Pockels coefficient[22,23]. We start with the Pockels tensor for LN because it contains the most elements, including all elements present in BTO. Fig. S6 illustrates the coordinate system (gray arrows) and the various angles that will be used in the following derivation.

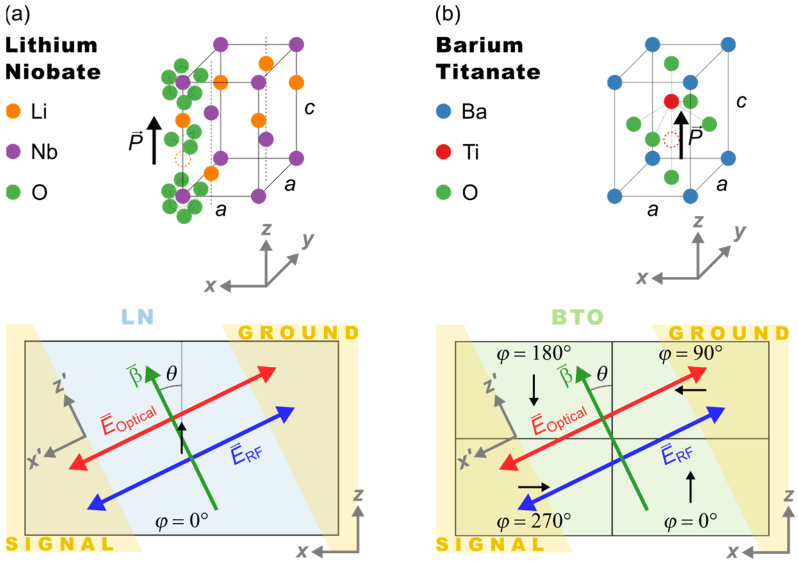

Fig. S6: Coordinate system for LN (a) and BTO (b) relative to the crystal axes. The original coordinate system is given by x, y, z, which are marked with the gray arrows. The rotated coordinate system corresponding to principal axes of the modified index ellipsoid under the influence of an external RF field is described with x', y', z'.

The refractive index in a crystal aligned where the x, y, z directions are aligned with the a, b, c crystal axes as in Fig. S6 can be described by an index ellipsoid[24]

$$1 = \left[\frac{1}{n_1^2} + \Delta\left(\frac{1}{n^2}\right)_1\right] x^2 + \left[\frac{1}{n_2^2} + \Delta\left(\frac{1}{n^2}\right)_2\right] y^2 + \left[\frac{1}{n_3^2} + \Delta\left(\frac{1}{n^2}\right)_3\right] z^2$$
$$+2\Delta\left(\frac{1}{n^2}\right)_4 yz + 2\Delta\left(\frac{1}{n^2}\right)_5 xz + 2\Delta\left(\frac{1}{n^2}\right)_6 xy\,. \tag{15}$$

The change in refractive index along a given dimension is given by[24]

$$\Delta\left(\frac{1}{n^2}\right)_{\text{ij}} = \sum_{\text{k}=1}^{3} r_{\text{ijk}} E_{\text{RF,k}}\ , \tag{16}$$

where $E_{\text{RF,k}}$ is the RF electric field along direction $\text{k} = x, y, z$. The matrix form including the electro-optic coefficients of LN is given by

$$\begin{bmatrix} \Delta(1/n^2)_1 \\ \Delta(1/n^2)_2 \\ \Delta(1/n^2)_3 \\ \Delta(1/n^2)_4 \\ \Delta(1/n^2)_5 \\ \Delta(1/n^2)_6 \end{bmatrix} = \begin{bmatrix} 0 & -r_{22} & r_{13} \\ 0 & r_{22} & r_{13} \\ 0 & 0 & r_{33} \\ 0 & r_{42} & 0 \\ r_{42} & 0 & 0 \\ -r_{22} & 0 & 0 \end{bmatrix} \begin{bmatrix} E_{\text{RF},x} \\ E_{\text{RF},y} \\ E_{\text{RF},z} \end{bmatrix}, \tag{17}$$

where we have used the reduced Voigt notation for indices ij. The situation for BTO is obtained by setting r_{22} = 0. Carrying out the matrix multiplication leads to

$$\begin{bmatrix} \Delta(1/n^2)_1 \\ \Delta(1/n^2)_2 \\ \Delta(1/n^2)_3 \\ \Delta(1/n^2)_4 \\ \Delta(1/n^2)_5 \\ \Delta(1/n^2)_6 \end{bmatrix} = \begin{bmatrix} r_{13}E_{\mathrm{RF},z} - r_{22}E_{\mathrm{RF},y} \\ r_{13}E_{\mathrm{RF},z} + r_{22}E_{\mathrm{RF},y} \\ r_{33}E_{\mathrm{RF},z} \\ r_{42}E_{\mathrm{RF},y} \\ r_{42}E_{\mathrm{RF},x} \\ -r_{22}E_{\mathrm{RF},y} \end{bmatrix} . \tag{18}$$

Now that we have the $\Delta(1/n^2)$ for each set of ij indices, the index ellipsoid can be written in the form

$$\begin{aligned} 1 = {} & \left(\frac{1}{n_o^2} - r_{22}E_{\mathrm{RF},y} + r_{13}E_{\mathrm{RF},z}\right)x^2 + \left(\frac{1}{n_o^2} + r_{22}E_{\mathrm{RF},y} + r_{13}E_{\mathrm{RF},z}\right)y^2 \\ & + \left(\frac{1}{n_e^2} + r_{33}E_{\mathrm{RF},z}\right)z^2 \\ & + (r_{42}E_{\mathrm{RF,y}})2yz + (r_{42}E_{\mathrm{RF,x}})2xz - (r_{22}E_{\mathrm{RF},y})2xy \ . \end{aligned} \tag{19}$$

In our case, the ordinary axis is the *a*-axis with $n_1 = n_2 = n_o$ and the extraordinary axis is the *c*-axis with $n_3 = n_e$. For the *a*-axis substrate the *z*- and *x*-axes lie in-plane and the y-axis is out of the plane. The waveguide is oriented along the *z*-axis and both the optical and external fields are polarized along the *x*-axis when θ = 0. To find the optimum angle θ we first apply a coordinate rotation around the *y*-axis to get new axes z' and x'

$$\begin{pmatrix} x \\ z \end{pmatrix} = \begin{pmatrix} \cos\theta & \sin\theta \\ -\sin\theta & \cos\theta \end{pmatrix} \begin{pmatrix} x' \\ z' \end{pmatrix} . \tag{20}$$

The axes and the electric fields can be expressed as

$$\begin{aligned} x &= x'\cos\theta + z'\sin\theta \\ y &= y' \\ z &= z'\cos\theta - x'\sin\theta \\ E_{\mathrm{RF},x} &= E_{\mathrm{RF,x'}}\cos\theta \\ E_{\mathrm{RF},z} &= -E_{\mathrm{RF},z'}\sin\theta \ . \end{aligned} \tag{21}$$

Here we have omitted electric field terms in the y' and z' directions because both optical and RF fields in the EO region of the phase shifter are only in the x' direction. Each of these terms is inserted into the index ellipsoid. After expanding all multiplication terms, we can drop all terms except those that contain $x'x' = x'^2$ because we are confined to the case where the optical and RF fields are both polarized along the x' direction. The resulting index ellipsoid is reduced to

$$\begin{aligned} 1 = \Bigg[& \frac{\cos^2\theta}{n_o^2} + \frac{\sin^2\theta}{n_e^2} - r_{13}E_{\mathrm{RF,x'}}\cos^2\theta\sin\theta - r_{33}E_{\mathrm{RF,x'}}\sin^3\theta \\ & - 2r_{42}E_{\mathrm{RF,x'}}\cos^2\theta\sin\theta \Bigg] x'^2 \ . \end{aligned} \tag{22}$$

The refractive index along the $x'x'$ direction is then given by

$$\begin{aligned} \frac{1}{n_{x'x'}^2} = \Bigg[& \frac{\cos^2\theta}{n_o^2} + \frac{\sin^2\theta}{n_e^2} - (r_{13} + 2r_{42})E_{\mathrm{RF,x'}}\cos^2\theta\sin\theta \\ & - r_{33}E_{\mathrm{RF,x'}}\sin^3\theta \Bigg] . \end{aligned} \tag{23}$$

In the absence of an external field, the refractive index of the $x'x'$-polarized wave is a combination of n_o and n_e

$$\frac{1}{n^2_{x'x'}}(\theta) = \frac{\cos^2\theta}{n_o^2} + \frac{\sin^2\theta}{n_e^2} \quad \text{or} \quad n_{x'x'}(\theta) = \frac{n_o n_e}{\sqrt{n_o^2\sin^2\theta + n_e^2\cos^2\theta}} \ . \tag{24}$$

In the presence of an external field $E_{\Omega,\mathrm{x}'}$ the remaining terms give the change in refractive index

$$\Delta\left(\frac{1}{n^2_{x'x'}}\right) = -E_{\mathrm{RF,x}'}[(r_{13} + 2r_{42})\cos^2\theta\sin\theta + r_{33}\sin^3\theta] \ . \tag{25}$$

The term in brackets is what determines the effective Pockels coefficient r_{eff} which in this case is equivalent to $r_{x'x'}$.

$$r_{\mathrm{eff}}(\theta) = (r_{13} + 2r_{42})\cos^2\theta\sin\theta + r_{33}\sin^3\theta \tag{26}$$

For BTO, one must also consider the effect of multiple domains oriented at 90° to one another. For this, we introduce φ to describe the domain orientation (φ = 0°, 90°, 180°, 270°) as well as v_φ to describe the fraction of domains with orientation φ ($0 \leq v_\varphi \leq 1$ and $\sum_\varphi v_\varphi = 1$). The effective Pockels coefficient of the multi-domain film is then the weighted average of $r_{\mathrm{eff}}(\theta + \varphi)$ over all domain orientations φ with weights given by v_φ.

$$r_{\mathrm{eff}}(\theta) = \sum_\varphi v_\varphi(\cos^2(\theta+\varphi)\sin(\theta+\varphi)\,(r_{13} + 2r_{42}) + r_{33}\sin^3(\theta+\varphi)) \tag{27}$$

3.1.1 Angular Dependence of the Permittivity

The permittivity in the BTO film has no angular dependence because it is an average across many domains oriented at right angles to each other. In a single domain, the angular dependence of the refractive index can be described by the radius of the ellipse with semi-axes corresponding to the refractive index along each crystal axis

$$n^2(\theta) = n_c^2\sin^2\theta + n_a^2\cos^2\theta \tag{28}$$

The net refractive index of the film is the average across all domains. The refractive index is the same for anti-parallel domains so only 90-degree domains need to be considered. The net refractive index is then

$$\begin{aligned} n^2(\theta) &= \frac{1}{2}[(n_c^2\sin^2\theta + n_a^2\cos^2\theta) + (n_a^2\sin^2\theta + n_c^2\cos^2\theta)] \\ n^2(\theta) &= \frac{(n_a^2 + n_c^2)}{2}[\sin^2\theta + \cos^2\theta] = \frac{(n_a^2 + n_c^2)}{2} \end{aligned} \tag{29}$$

With $\varepsilon = n^2$, the permittivity is $\varepsilon = \frac{1}{2}(\varepsilon_a + \varepsilon_c)$. Thus, there is no angular dependence for multi-domain films.

3.2 Fitting $\boldsymbol{r}_{\mathbf{ij}}$ Coefficients to the Measured $\boldsymbol{r}_{\mathbf{eff}}$ Data

In this section we describe the fitting process that was used to extract the individual Pockels tensor elements from the measurements of the effective Pockels coefficient. While the effective Pockels coefficient is useful as a single number that quantifies the EO strength in a device, it is still of interest to know the values of the individual elements that comprise the Pockels tensor. The angular dependence of r_{eff} provides a framework to do this. Equation (27) indicates a dependence on θ with coefficients $r_{13} + 2r_{42}$ and r_{33}. This assumes fixed values of φ and v_φ which is the case for our measurements since we initially apply a DC bias and then remove it before allowing the films to reach an equilibrium state

for the domain fractions (see Supplementary Note S2.4). For LN we have $\nu_{\varphi=0^\circ}$ = 1 because it is a single domain film. For BTO we use the partial-poled cased described above with $\nu_{\varphi=0^\circ,90^\circ}$ = 0.375 and $\nu_{\varphi=180^\circ,270^\circ}$ = 0.125. These domain fractions best represent the situation where the domain fractions are allowed to reach an equilibrium state after an initial poling step, which is described in the following section[25].

If one has a collection of $r_{\text{eff}}(\theta)$ measurement points from enough angles, then this equation can be fit to the data by using the aforementioned coefficients as fitting parameters. In this way, individual Pockels tensor elements can be determined rather than just r_{eff}. We take $r_{\text{eff}}(\theta, p_1, p_2)$ where θ is the independent variable and the new parameters are $p_1 = r_{13} + 2r_{42}$ and $p_2 = r_{33}$. Fig. S7 shows the measured $r_{\text{eff}}(\theta)$ points at a frequency of 10 GHz as well as the fitted curve according to equation (27). The LN data in Fig. S7 was fitted with values of p_1 = 28.9 ± 2.0 pm/V and p_2 = 26.9 ± 0.9 pm/V while the BTO data was fitted values of p_1 = 838 ± 29 pm/V and p_2 = 64.8 ± 16.2 pm/V. For LN which is a single domain thin film, there is only one non-zero domain fraction $\nu_{\varphi=0^\circ}$ = 1. For BTO we used domain fractions of $\nu_{\varphi=0^\circ,90^\circ}$ = 0.375 and $\nu_{\varphi=180^\circ,270^\circ}$ = 0.125, corresponding to the equilibrium domain fractions as explained in the main text. The domain fractions for BTO lead to an equation (27) that has symmetry every 45°. For LN, the resulting form of equation (27) leads to symmetry for every 90°. The LN devices with θ < 20° had modulation signals that were too weak measure, because the effective Pockels coefficient at these angles is so small.

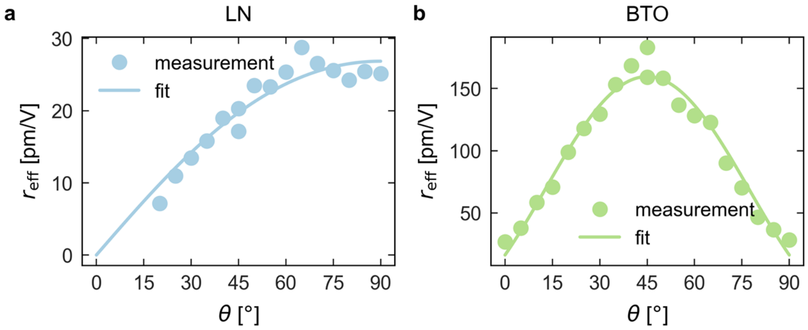

Fig. S7 | Angular dependence of the effective Pockels coefficient. Measured effective Pockels coefficients (circles) and the resulting fit (line) of equation (27) to the measurement points for **a** LN and **b** BTO. The plots show the results measured at a modulation frequency of 10 GHz. This process was repeated for each frequency point the get the results in Fig. 4 of the main text.

3.3 Poling Dynamics

Here we discuss the procedure for aligning the ferroelectric polarization of a majority of BTO domains in the same orientation to achieve a non-zero net EO effect (see the discussion of random vs. partial-poled vs. poled in the main text). Note that LN does not require poling because the thin film is already in a poled, single-domain state.

For each BTO device an initial poling procedure was performed where a DC bias was applied in addition to a 50 GHz modulation signal. The difference in dB between the powers in the first modulation sideband and in the optical carrier (peak-sideband ratio) was monitored while the magnitude of the DC bias was increased in 1 V increments. The sideband powers increase with the bias voltage as BTO domains start to flip polarizations. Eventually the peak-sideband ratio saturates at a maximum when no more domain polarizations are able to switch even with further increases in the bias. This corresponds to the "poled" state. Ideally, all electrical and EO measurements would be performed

under this maximum bias, however, this was not possible for frequencies above 110 GHz due to available equipment. For consistency across all frequencies, all measurements were therefore performed without a DC bias.

To ensure a stable domain polarization state, the time decay of the peak-sideband ratio was measured. When the bias is removed, domains start to randomly flip to the opposite polarization and the peak-sideband ratio immediately decrease. Fig. S8 shows the decay in the peak-sideband ratio (i.e. modulation efficiency) over time and measured from the instant that the bias was removed. The modulation efficiency eventually settles to a value around 1 dB less than its initial value. This corresponds to the "partial-poled" state, where a majority of domains are still aligned in a preferred direction. All electrical and EO measurements were performed after this poling and decay sequence.

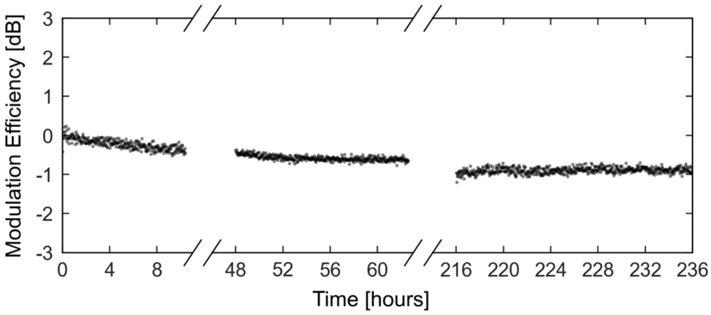

Fig. S8 | Time decay of modulation efficiency. Modulation efficiency as a function of time since the initial DC bias was removed that was used to pole the BTO film. The modulation efficiency stabilizes at a value only 1 dB less than the maximum efficiency with the DC bias.

4 Derivation of the Dependence of $\boldsymbol{\Delta n}$ on $\boldsymbol{\varepsilon_{\mathrm{BTO}}}$

This section provides a derivation for equations (6-8) in the main text, which are analytical formulas for the refractive index change of an EO material in the context of the hypothetical structure in Fig. S9.

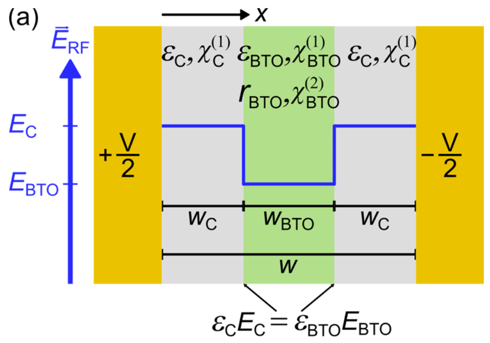

Fig. S9 | Hypothetical phase shifter used to find an analytical dependence of the refractive index change in BTO layer on the susceptibility or permittivity of the materials in the structure. A potential difference between metal electrodes denoted with G and S results in an electric field $\vec{E}_{\mathrm{RF}}$ (blue arrow) across the layers. The middle layer in green is BTO. It is separated from the electrodes by a cladding material (gray).

The Pockels effect depends on the electric field E_{BTO} in the region of the BTO. Therefore, we start by looking for an expression for E_{BTO} that depends on the permittvities $\varepsilon_{\mathrm{BTO}}$ and ε_{C} as well as the layer widths w_{BTO} and w_{C}. The potential difference V between the ground and signal electrodes is

$$\begin{aligned} V &= \int_0^{2w_{\mathrm{C}}} E_{\mathrm{C}} dx + \int_0^{w_{\mathrm{BTO}}} E_{\mathrm{BTO}} dx \\ &= 2w_{\mathrm{C}} E_{\mathrm{C}} + w_{\mathrm{BTO}} E_{\mathrm{BTO}} \ . \end{aligned} \tag{30}$$

Rearranging for E_{BTO} gives

$$E_{\mathrm{BTO}} = \frac{V - 2w_{\mathrm{C}} E_{\mathrm{C}}}{w_{\mathrm{BTO}}} = \frac{V}{w_{\mathrm{BTO}}} - \frac{2w_{\mathrm{C}} E_{\mathrm{C}}}{w_{\mathrm{BTO}}} \ . \tag{31}$$

Let $R_w = 2w_{\mathrm{C}}/w_{\mathrm{BTO}}$ be the ratio of the total width of the cladding material to the width of the EO such that the total width w between the electrodes can be expressed as $w = 2w_{\mathrm{C}} + w_{\mathrm{BTO}} = w_{\mathrm{BTO}}(1 + R_w)$. This allows equation (31) to be rewritten as

$$\begin{aligned} E_{\mathrm{BTO}} &= \frac{V}{w}(1 + R_w) - R_w E_{\mathrm{C}} \\ &= \frac{V}{w}(1 + R_w) - R_w \frac{\varepsilon_{\mathrm{BTO}}}{\varepsilon_{\mathrm{C}}} E_{\mathrm{BTO}} \ , \end{aligned} \tag{32}$$

where E_{C} was eliminated by using the boundary condition $\varepsilon_{\mathrm{C}} E_{\mathrm{C}} = \varepsilon_{\mathrm{BTO}} E_{\mathrm{BTO}}$ for electric fields that are perpendicular to an interface between two materials. Equation (32) can then be solved to find the expression for E_{BTO} that was sought from the start

$$E_{\mathrm{BTO}} = \frac{V}{w} \frac{1 + R_w}{1 + R_w \frac{\varepsilon_{\mathrm{BTO}}}{\varepsilon_{\mathrm{C}}}} \ . \tag{33}$$

In the second step we relate the first and second order susceptibilities. Miller's rule in equation (18) of the main text provides this relation

$$\delta = \frac{\chi^{(2)}(\omega_0 \pm \omega_m, \omega_0, \omega_m)}{\chi^{(1)}(\omega_0 \pm \omega_m)\chi^{(1)}(\omega_0)\chi^{(1)}(\omega_m)} \ . \tag{34}$$

The optical frequencies are much larger than the modulating frequencies so $\omega_0 \pm \omega_m \approx \omega_0$ and $\chi^{(1)}(\omega_0 \pm \omega_m) \approx \chi^{(1)}(\omega_0)$. The second order susceptibility as a function of the first order RF susceptibility is then

$$\chi^{(2)}(\omega_0 \pm \omega_m, \omega_0, \omega_m) = \delta \left(\chi^{(1)}(\omega_0)\right)^2 \chi^{(1)}(\omega_m) \ . \tag{35}$$

For the final step we find an expression for the change of refractive index in the electro-optic material Δn that depends on $\varepsilon_{\mathrm{BTO}}$ rather than on r_{BTO}. Start with the common expression for Δn given by

$$\Delta n = -\frac{1}{2} n_0^3 r_{\mathrm{BTO}} E_{\mathrm{BTO}} \ . \tag{36}$$

where n_0 represents the value of the unperturbed optical refractive index. Using the fact that $r_{\mathrm{BTO}} = -2\chi^{(2)}_{\mathrm{BTO}}/n^4$, where $\chi^{(2)}_{\mathrm{BTO}} = \chi^{(2)}(\omega_0 \pm \omega_m, \omega_0, \omega_m)$, we can write

$$\Delta n = \frac{\chi^{(2)}_{\mathrm{BTO}}}{n_0} E_{\mathrm{BTO}} \ . \tag{37}$$

Now inserting the expressions of equation (33) and equation (35) gives

$$\Delta n = \frac{1}{n_0}\left[\delta \left(\chi^{(1)}(\omega_0)\right)^2 \chi^{(1)}_{\mathrm{BTO}}(\omega_m)\right]\left[\frac{V}{w}\frac{1+R_w}{1+R_w\frac{\varepsilon_{\mathrm{BTO}}}{\varepsilon_{\mathrm{C}}}}\right] \ . \tag{38}$$

We would like the expression to be normalized to a given V/w. Also, the optical refractive index n_0 and susceptibility $\chi^{(1)}(\omega_0)$ are taken to be constant. These terms are therefore grouped with δ into a single constant η such that

$$\Delta n = \eta \frac{(1+R_w)\chi^{(1)}_{\mathrm{BTO}}(\omega_m)}{1+R_w\frac{\varepsilon_{\mathrm{BTO}}}{\varepsilon_{\mathrm{C}}}},$$
$$\eta = \delta \frac{\left(\chi^{(1)}(\omega_0)\right)^2}{n_0}\frac{V}{w} = \delta \frac{(n_0^2-1)^2}{n_0}\frac{V}{w}. \tag{39}$$

Finally, we use the fact that $\chi^{(1)}_{\mathrm{BTO}}(\omega_m) = \varepsilon_{\mathrm{BTO}} - 1$ to arrive at the expression in equation (6) of the main text

$$\Delta n = \eta \frac{(1+R_w)(\varepsilon_{\mathrm{BTO}}-1)}{1+R_w\frac{\varepsilon_{\mathrm{BTO}}}{\varepsilon_{\mathrm{C}}}} \ . \tag{40}$$

In the limit of small R_w (i.e. plasmonic devices, w_{C} = 0), equation (40) can be simplified to equation (7) in the main text

$$\Delta n_{\mathrm{BTO}}(R_w = 0) = \eta(\varepsilon_{\mathrm{BTO}} - 1) \ . \tag{41}$$

In the limit of large R_w (i.e. photonic devices, $w_{\mathrm{C}} \gg w_{\mathrm{BTO}}$), equation (40) can be simplified to the approximation given in equation (8) in the main text

$$\Delta n_{\mathrm{BTO}}(R_w \gg 1) \approx \eta \varepsilon_{\mathrm{C}} \frac{\varepsilon_{\mathrm{BTO}} - 1}{\varepsilon_{\mathrm{BTO}}}. \tag{42}$$

5 Voltage-Length Product Dependencies

In this section we discuss the dependence of the phase shifter's voltage-length product $V_\pi L$ on various structural parameters and the modulation frequency, as calculated from simulations.

5.1 Varying Ferroelectric Permittivity

Here we discuss the $V_\pi L$ dependence on the permittivity of the EO layer $\varepsilon_{\mathrm{EO}}$ and the modulation frequency. The last section of the main text describes the relationship between the Pockels shift and the permittivity of the EO material. Specifically, it discusses how the RF electric field is pushed out of the EO layer when $\varepsilon_{\mathrm{EO}}$ is large, which should reduce the modulator's efficiency. In Fig. S10(a) we show this effect for the phase shifter in this work. The $V_\pi L$ is calculated from simulations according to the procedure described in the methods section. The effective Pockels coefficient is held constant for all $\varepsilon_{\mathrm{EO}}$ to highlight the effect of the changing RF electric field distribution. A r_{eff} of 440 pm/V was chosen since it is the maximum value of r_{eff} in the fully poled case (see Fig. 3, main text). Fig. S10(a) shows that the $V_\pi L$ increases (i.e. modulator becomes less efficient) for higher $\varepsilon_{\mathrm{EO}}$ as a result of the smaller RF electric field in the EO region. This result also highlights the importance of having accurate permittivity measurements when it comes to measuring $V_\pi L$ and Pockels coefficients.

Simulations of the $V_\pi L$ also show that there is little dependence on the modulation frequency in the range of interest to this work. Fig. S10(b) shows this dependence from 100 MHz to 500 GHz for various values of $\varepsilon_{\mathrm{EO}}$. This indicates that the cross-section of the phase shifter is small enough relative to the RF wavelengths such that the RF electric field distribution is not changing with frequency. In other words, the quasi-static TEM assumption that is assumed for the conformal mapping equations in section 2 above seems to hold up to at least 500 GHz.

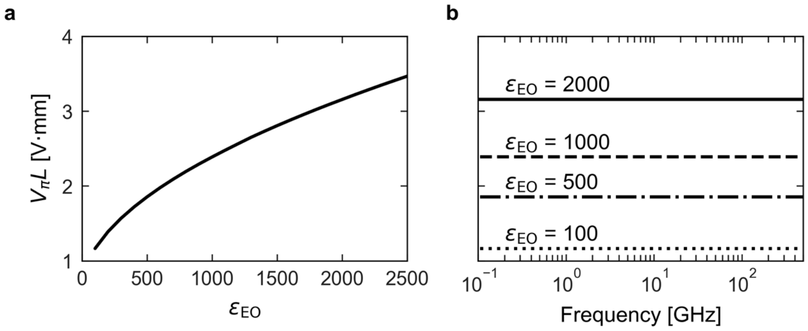

Fig. S10 | Dependence of the voltage-length product on permittivity and frequency. a, Simulated voltage-length product of the phase shifter in this work for various values of the EO layer's permittivity and assuming a constant r_{eff} = 440 pm/V. **b**, Simulated voltage-length product of the phase shifter in this work as a function of modulation frequency and for various values of EO layer permittivity.

5.2 Varying Layer Thicknesses

The main text alluded to the phase shifters in this work having an optimal design for EO characterization. Part of the reason for this is the reproducibility of the fabrication process and the minimal uncertainty in the dimensions of the devices under test. One of the few areas of uncertainty that can influence the measured results is the thickness of the EO and oxide layers. Fig. S11(a) shows the simulated dependence of the phase shifter's voltage-length product on the error in height of the EO layer Δh_{EO}. The $V_\pi L$ becomes

smaller for negative Δh_{EO} (i.e. smaller h_{EO}) due to changes in the distributions of the optical and RF modes. Simulations were performed with different values for the permittivity of the EO layer $\varepsilon_{\mathrm{EO}}$ to account for the permittivity dispersion of BTO, however, this has little influence. Two values of $\varepsilon_{\mathrm{EO}}$ are plotted in Fig. S11 to show this, roughly corresponding to the values for LN ($\varepsilon_{\mathrm{EO}}$ = 30, blue) and BTO at low frequency ($\varepsilon_{\mathrm{EO}}$ = 2500, green). The data is plotted across a range covering Δh_{EO} = ±10 %, but this is far greater than the actual uncertainty in thickness across the dimensions of a small chip like the ones used in this work. As an example, the BTO used in this work had a thickness of 485 nm. Since the BTO was grown epitaxially and its unit cell is roughly 0.4 nm, the typical error in thickness is on the order of just a few nanometers, which is less than 1 % of the total film thickness. We therefore conclude that variation of h_{EO} in this type of phase shifter only influences the measured results by roughly 1 % at worst.

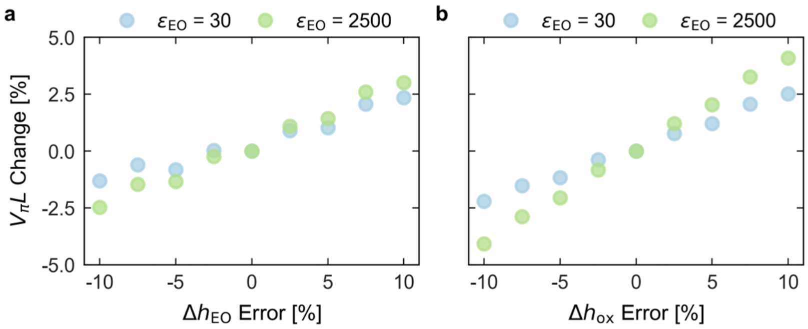

Fig. S11 | Influence of layer thicknesses on the voltage-length product. Simulated dependence of the voltage-length length product on the thickness of the **a** EO layer and **b** oxide spacer layer for the phase shifters in this work. The thicknesses are expressed as a percentage error from the designed value and the voltage-length products are expressed as a percentage change from the value at the designed thickness (i.e. Δh = 0). The dependence is given for two values of $\varepsilon_{\mathrm{EO}}$ roughly corresponding to the values for LN (30, blue) and BTO at low frequencies (2500, green).

The only other layer thickness with any non-negligible influence is that of the oxide layer h_{ox} between the EO layer and the electrodes. This, however, is even more inconsequential than variation in h_{EO} as shown in Fig. S11(b). The effect of Δh_{ox} follows the same trend as that of Δh_{EO}. This is because the RF electric field is typically stronger in the EO layer for smaller h_{ox} which leads to smaller $V_{\pi}L$. The oxide layers in this work were deposited with PECVD after calibrating the deposition rate such that the error in thickness was less than 5 nm for 100 nm of oxide. We can similarly conclude that variation of h_{ox} has an influence on the measured results of less than a few percent.

References

1. Wemple, S. H. & DiDomenico, M. Electrooptical and Nonlinear Optical Properties of Crystals. in *Applied Solid State Science* (ed. Wolfe, R.) vol. 3 263–383 (Elsevier, 1972).
2. Dalton, L. R., Günter, P., Jazbinsek, M., Kwon, O.-P. & Sullivan, P. A. *Organic Electro-Optics and Photonics: Molecules, Polymers, and Crystals*. (Cambridge University Press, Cambridge, 2015). doi:10.1017/CBO9781139043885.
3. Burla, M. *et al.* 500 GHz plasmonic Mach-Zehnder modulator enabling sub-THz microwave photonics. *APL Photonics* **4**, 056106 (2019).
4. Salamin, Y. *et al.* Compact and ultra-efficient broadband plasmonic terahertz field detector. *Nat. Commun.* **10**, 5550 (2019).
5. Zgonik, M. *et al.* Dielectric, elastic, piezoelectric, electro-optic, and elasto-optic tensors of BaTiO3 crystals. *Phys. Rev. B* **50**, 5941–5949 (1994).
6. Jazbinšek, M. & Zgonik, M. Material tensor parameters of LiNbO3 relevant for electro- and elasto-optics. *Appl. Phys. B* **74**, 407–414 (2002).
7. Clemens, R., Luther, G. & Müser, H. E. Dielectric behaviour of ferroelectric barium titanate in the microwave region. *Phys. Status Solidi A* **64**, 637–645 (1981).
8. McNeal, M. P., Jang, S.-J. & Newnham, R. E. The effect of grain and particle size on the microwave properties of barium titanate (BaTiO3). *Journal of Applied Physics* **83**, 3288–3297 (1998).
9. Nakao, O., Tomomatsu, K., Ajimura, S., Kurosaka, A. & Tominaga, H. Influence of 180° domains on ferroelectric properties of BaTiO3 single crystal. *Appl. Phys. Lett.* **61**, 1730–1732 (1992).
10. Ummethala, S. *et al.* Hybrid electro-optic modulator combining silicon photonic slot waveguides with high-k radio-frequency slotlines. *Optica, OPTICA* **8**, 511–519 (2021).
11. Ummethala, S. Plasmonic-Organic and Silicon-Organic Hybrid Modulators for High-Speed Signal Processing. (2021). doi:10.5445/IR/1000136059.
12. Nassau, K., Levinstein, H. J. & Loiacono, G. M. Ferroelectric lithium niobate. 2. Preparation of single domain crystals. *J. Phys. Chem. Solids.* **27**, 989–996 (1966).
13. Ohmachi, Y., Sawamoto, K. & Toyoda, H. Dielectric Properties of LiNbO3 Single Crystal up to 9 Gc. *Jpn. J. Appl. Phys.* **6**, 1467 (1967).
14. Milanovic, V. *et al.* Characterization of broad-band transmission for coplanar waveguides on CMOS silicon substrates. *IEEE Transactions on Microwave Theory and Techniques* **46**, 632–640 (1998).
15. Yu, H. & Bogaerts, W. An Equivalent Circuit Model of the Traveling Wave Electrode for Carrier-Depletion-Based Silicon Optical Modulators. *Journal of Lightwave Technology* **30**, 1602–1609 (2012).
16. Chen, E. & Chou, S. Y. Characteristics of coplanar transmission lines on multilayer substrates: modeling and experiments. *IEEE Transactions on Microwave Theory and Techniques* **45**, 939–945 (1997).
17. Zhu, N. H., Pun, E. Y. B. & Li, J. X. Analytical formulas for calculating the effective dielectric constants of coplanar lines for OIC applications. *Microwave and Optical Technology Letters* **9**, 229–232 (1995).
18. Ghione, G. *et al.* Microwave modeling and characterization of thick coplanar waveguides on oxide-coated lithium niobate substrates for electrooptical applications. *IEEE Transactions on Microwave Theory and Techniques* **47**, 2287–2293 (1999).
19. Heinrich, W. Quasi-TEM description of MMIC coplanar lines including conductor-loss effects. *IEEE Transactions on Microwave Theory and Techniques* **41**, 45–52 (1993).
20. Kwon, Y. R., Hietala, V. M. & Champlin, K. S. Quasi-TEM Analysis of 'Slow-Wave' Mode Propagation on Coplanar Microstructure MIS Transmission Lines. *IEEE Transactions on Microwave Theory and Techniques* **35**, 545–551 (1987).
21. Jordan, E. C. & Balmain, K. G. *Electromagnetic Waves and Radiating Systems*. (Prentice-Hall, Inc., 1968).
22. Castera, P. *et al.* Electro-Optical Modulation Based on Pockels Effect in BaTiO3 With a Multi-Domain Structure. *IEEE Photonics Technology Letters* **28**, 990–993 (2016).

23. Messner, A. *et al.* Plasmonic Ferroelectric Modulators. *Journal of Lightwave Technology* **37**, 281–290 (2019).
24. Boyd, R. W. *Nonlinear Optics*. (Elsevier, 2008).
25. Abel, S. *et al.* Large Pockels effect in micro- and nanostructured barium titanate integrated on silicon. *Nat. Mater.* **18**, 42–47 (2019).
